# Gut microbiota and fecal metabolomic alterations in hospitalized patients with *Staphylococcus aureus* infection: an exploratory multi-omics study

**DOI:** 10.3389/fcimb.2026.1919715

**Published:** 2026-09-03

**Authors:** Yan Zhao, Wei Chen, Peiruo Chen, Xuxin Chen, Yiwei Ding, Zhihai Han

**Affiliations:** Department of Pulmonary and Critical Care Medicine, the Sixth Medical Center of Chinese PLA General Hospital, Beijing, China

**Keywords:** 16S rRNA sequencing, antimicrobial exposure, exploratory multi-omics, fecal metabolomics, gut microbiota, hospitalized patients, *Staphylococcus aureus*

## Abstract

**Introduction:**

This study characterized gut microbial composition and fecal metabolomic profiles in hospitalized patients with *Staphylococcus aureus* infection (SAI) and explored their associations with inflammatory and organ-function indicators.

**Methods:**

This single-center exploratory observational study included 16 hospitalized patients with microbiologically confirmed and clinically adjudicated SAI and 10 healthy controls (HCs). The first qualified fecal sample was collected within 7 days after microbiological confirmation. Fifteen patients had received antimicrobial treatment before sampling, whereas one had not; none of the HCs had received antibiotics before fecal collection. Gut microbiota were profiled by 16S rRNA sequencing, and fecal metabolites were analyzed by LC-MS/MS-based untargeted metabolomics. Genus-level differential abundance was assessed using ANCOM-BC2 with Benjamini-Hochberg correction and pseudo-count sensitivity analysis.

**Results:**

Alpha-diversity indices were lower in the SAI group. Bray-Curtis PERMANOVA showed a statistically significant but modest group-associated difference in community composition (F = 2.8654, R² = 0.1067, P = 0.0001), whereas PERMDISP was not significant (P = 0.0885). ANCOM-BC2 identified four genera with robustly higher bias-corrected abundance in the SAI group: *Corynebacterium*, the *[Clostridium] innocuum* group, *Enterococcus*, and *Eggerthella*. Their effect directions remained positive in culture-confirmed and respiratory-infection-only analyses, although not all retained pseudo-count-robust significance. Untargeted metabolomics detected 2,956 features, including 2,491 with putative MS/MS-based annotations. Using VIP > 1 and raw P < 0.05, 381 candidate features were identified. Exploratory enrichment signals involved tryptophan, glutathione, sulfur, bile acid, amino acid, and lipid metabolism, but were not regarded as FDR-confirmed. No clinical-omics association remained significant after FDR correction.

**Discussion:**

Hospitalized patients with SAI showed group-associated microbial and metabolic differences compared with HCs. However, because nearly all patients were sampled after antimicrobial exposure, these patterns cannot be separated from treatment, hospitalization, disease severity, and other clinical interventions. The findings are therefore preliminary and hypothesis-generating rather than *S. aureus*-specific signatures.

## 1 Introduction

*Staphylococcus aureus* is a clinically important pathogen that can cause skin and soft-tissue infection, pneumonia, bloodstream infection, infective endocarditis, sepsis, and multiple-organ dysfunction (Thwaites et al., 2011; Tong et al., 2015; van Hal et al., 2012). Previous research has largely focused on *S. aureus* virulence factors, antimicrobial resistance, host inflammatory responses, and vascular endothelial injury.

The gut microbiota is an important determinant of immune homeostasis, intestinal-barrier integrity, and metabolic balance. Specific commensal taxa and microbially derived metabolites have been implicated in anti-inflammatory activity and maintenance of intestinal-barrier function (Sokol et al., 2008; Venkatesh et al., 2014), while population-based evidence indicates that microbiome composition is also influenced by numerous host and environmental factors (Zhernakova et al., 2016). Infection, critical illness, antimicrobial exposure, hospitalization, altered nutritional intake, and systemic inflammation may collectively perturb microbial community structure. Recent clinical multi-omics studies in sepsis have further demonstrated that microbial and fecal metabolic profiles may vary according to disease severity and clinical outcome (Sun et al., 2023). Recent reviews have also emphasized the reciprocal relationships among intestinal dysbiosis, antimicrobial treatment, host inflammation, and organ dysfunction during severe infection (Piccioni et al., 2024; Tao et al., 2024).

The relationship between *S. aureus* infection and the intestinal microbiota remains incompletely characterized. A recent review summarized emerging evidence linking *S. aureus* colonization or infection with intestinal microbial ecology and highlighted the need to distinguish pathogen-associated signals from treatment-related and host-related effects (Liu et al., 2024). Experimental research has also suggested that gut-lung microbial and metabolic interactions may influence host responses during MRSA pneumonia, although such mechanistic findings cannot be directly extrapolated to the present clinical cohort (Zhao et al., 2023).

Compared with microbial compositional profiling alone, integrated fecal metabolomics may provide complementary information regarding the local intestinal metabolic environment. However, observational associations among microbial taxa, metabolites, and clinical indicators cannot establish microbial production pathways or causal effects.

This study aimed to integrate 16S rRNA sequencing, untargeted fecal metabolomics, and clinical phenotype data to describe gut microbial composition and fecal metabolic profiles in hospitalized patients with clinically adjudicated SAI and to explore their covariance with inflammatory and organ-function indicators.

## 2 Materials and methods

### 2.1 Study population and clinical data collection

This single-center exploratory observational study enrolled patients with microbiologically confirmed *Staphylococcus aureus* infection and healthy controls. Participants were assigned to the healthy control group or the *S. aureus* infection group. Fecal samples were collected from both groups for 16S rRNA sequencing and untargeted fecal metabolomic analysis. A total of 26 fecal samples were included in both analyses, comprising 10 samples from healthy controls and 16 samples from patients with SAI.

The inclusion criteria for patients with SAI were as follows: age 18 years or older; microbiological detection of *S. aureus* from a clinically relevant specimen together with a standardized clinical adjudication supporting active infection rather than colonization or contamination; availability of a qualified fresh fecal sample collected within 7 days after microbiological confirmation; and availability of clinical data suitable for the analysis of inflammatory and organ-function indicators.

Healthy controls were recruited from individuals without acute infection, recent hospitalization, active gastrointestinal disease, or other clinically apparent acute illnesses. None of the healthy controls had received antibiotic treatment before fecal sample collection. Healthy controls were excluded if they had active gastrointestinal infection, inflammatory bowel disease, gastrointestinal involvement by malignancy, or recent use of probiotics or other microbiota-modulating preparations.

Age and sex were recorded for all participants. Disease-related laboratory indicators, severity scores, organ-support measures, complications, and clinical outcomes were collected only for patients with SAI. Detailed information regarding body mass index, chronic comorbidities, dietary intake, and non-antimicrobial concomitant medication exposure was not systematically available for the healthy controls. Formal between-group comparisons were therefore limited to age and sex, and multivariable adjustment for other baseline differences was not performed.

The clinical relevance of each positive *S. aureus* result was independently reviewed by two physicians specializing in pulmonary and critical care medicine using predefined adjudication criteria. Disagreements were resolved by consensus with a senior investigator. The adjudication incorporated the anatomical infection site, specimen type and quality, compatible clinical manifestations, inflammatory indicators, radiological findings, microbiological results, potential coinfections, and the final diagnosis documented by the treating clinical team. For respiratory tract cases, a positive sputum culture or detection of *S. aureus* in bronchoalveolar lavage fluid by next-generation sequencing alone was not considered sufficient to establish active infection. Respiratory SAI required compatible respiratory manifestations and imaging findings together with microbiological evidence. Cases considered more consistent with airway colonization, specimen contamination, or an alternative primary infection were excluded.

Based on this clinical adjudication, infections were categorized as respiratory tract infections or bloodstream infections. Microbiological confirmation was obtained using sputum culture, blood culture, or detection of *S. aureus* in bronchoalveolar lavage fluid by next-generation sequencing. Potential bacterial, fungal, and viral coinfections were reviewed from the available clinical microbiology records and recorded in the patient-level supplementary metadata.

Bloodstream infection was defined as the detection of *S. aureus* in blood culture together with a compatible clinical diagnosis. The duration of bacteremia was not calculated because serial blood-culture clearance dates were not consistently available.

To further evaluate the clinical relevance of gut microbial alterations, clinical data were collected from the 16 patients with SAI, including age, sex, body mass index, hospital location, infection category, microbiological confirmation method, antibiotic treatment, white blood cell count, neutrophil count, C-reactive protein, procalcitonin, creatinine, albumin, lactate, APACHE II score, SOFA score, organ-support measures, complications, and clinical outcomes.

These variables were used to describe infection-related inflammatory burden, organ-function status, disease severity, nutritional or inflammation-related depletion, clinical interventions, and prognosis. Because C-reactive protein, procalcitonin, and lactate were non-normally distributed, subsequent descriptive and correlation analyses were performed using nonparametric methods. For the clinical–omics analysis, laboratory indicators and disease-severity variables obtained within 24 h of fecal sample collection were used. When more than one measurement was available within this window, the measurement temporally closest to fecal collection was selected. Values unavailable within the predefined 24-h window were treated as missing and were not imputed. SOFA and APACHE II scores used in the integrated analysis were based on the corresponding clinical assessment within the same 24-h sampling window. Clinical outcomes were recorded separately over the subsequent hospitalization and were not treated as concurrent measurements at fecal sampling. Sepsis and septic shock were adjudicated using the Sepsis-3 framework (Singer et al., 2016; Shankar-Hari et al., 2016). Sepsis was defined as infection accompanied by an acute increase in SOFA score of at least 2 points from the estimated preinfection baseline. Septic shock was treated as a subset of sepsis and was defined by a requirement for vasopressor therapy to maintain a mean arterial pressure of at least 65 mmHg and a serum lactate concentration greater than 2 mmol/L despite adequate fluid resuscitation. Baseline SOFA was determined from available preinfection clinical information, and an observed SOFA score of at least 2 was not used alone to establish sepsis.

Age and sex were available for both groups and were formally compared between the HC and SAI cohorts. Disease-related laboratory indicators, severity scores, organ-support measures, complications, and clinical outcomes were collected only for patients with SAI. Detailed body mass index, chronic-comorbidity, dietary, and concomitant-medication information was not systematically available for the healthy controls. Therefore, formal between-group comparisons were limited to age and sex, and multivariable adjustment for other baseline differences was not performed.

Pre-sampling antimicrobial exposure was abstracted from the prospectively collected case-record forms and medication records. Fifteen of the 16 patients with SAI had received antimicrobial treatment before fecal sample collection, whereas one patient had an explicit record indicating no antimicrobial treatment before sampling. None of the healthy controls had received antibiotics before fecal sample collection. The specific antimicrobial agents administered before fecal sampling were recorded at the patient level and are provided in **Supplementary Table 1**. Glucocorticoids, including hydrocortisone and dexamethasone, were not classified as antimicrobial agents and were recorded separately.

Although the temporal ordering of antimicrobial administration and fecal sampling was confirmed, the exact interval between antimicrobial initiation and fecal collection, treatment duration, dosage, and cumulative pre-sampling antimicrobial exposure could not be consistently quantified for all patients. Because only one patient with SAI was unexposed before sampling, a formal comparison between antimicrobial-exposed and antimicrobial-unexposed patients and multivariable adjustment for antimicrobial exposure were not statistically feasible.

Antimicrobial exposure, hospitalization duration, nutritional intake, underlying diseases, infection site, glucocorticoid exposure, organ-support measures, and other clinical interventions were considered major potential confounders when interpreting the microbiome and metabolomic findings. All microbiome–metabolome–clinical phenotype associations were therefore interpreted as exploratory and hypothesis-generating.

### 2.2 Fecal sample collection and storage

For patients with SAI, the first available qualified fecal sample was collected within 7 days after microbiological confirmation of *S. aureus* infection. Because fecal collection depended on spontaneous bowel movements, samples were not obtained on an identical prespecified day after microbiological confirmation. Fifteen of the 16 patients with SAI had received antimicrobial treatment before fecal collection, whereas one patient had not received antimicrobial treatment before sampling.

The date and time of fecal collection were recorded and were used to define the corresponding 24-h clinical-data window. However, the exact elapsed interval between microbiological confirmation and fecal collection was not available as a sufficiently complete and standardized patient-level variable for quantitative analysis. Similarly, although antimicrobial exposure preceded sampling in 15 patients, the exact treatment-to-sampling interval could not be consistently quantified.

For healthy controls, fecal samples were collected at study enrollment, and none had received antibiotics before sampling. Fresh fecal samples were collected into sterile sampling tubes, transported to the laboratory under low-temperature conditions as soon as possible, and stored at −80 °C until analysis. Aliquots from each fecal sample were used for microbial DNA extraction and untargeted metabolomic profiling. Repeated freeze–thaw cycles were avoided before analysis.

### 2.3 16S rRNA sequencing and bioinformatic analysis

Total microbial genomic DNA was extracted from fecal samples using the TGuide S96 Magnetic Soil/Stool DNA Kit (Tiangen Biotech Co., Ltd., Beijing, China) according to the manufacturer’s instructions. DNA integrity was evaluated by electrophoresis on a 1.8% agarose gel, and DNA concentration and purity were assessed using a NanoDrop 2000 UV–Vis spectrophotometer (Thermo Fisher Scientific, Wilmington, DE, USA).

The V3–V4 hypervariable region of the bacterial 16S rRNA gene was amplified using the forward primer 338F (5′-ACTCCTACGGGAGGCAGCA-3′) and the reverse primer 806R (5′-GGACTACHVGGGTWTCTAAT-3′). Sample-specific Illumina index sequences were incorporated into the forward and reverse primers.

PCR amplification was performed in a 20-μL reaction mixture containing 5–50 ng of template DNA, 0.3 μL of each primer (10 μM), 5 μL of KOD FX Neo Buffer, 2 μL of dNTP mixture (2 mM each), 0.2 μL of KOD FX Neo polymerase, and nuclease-free water to a final volume of 20 μL. The thermal-cycling conditions consisted of an initial denaturation at 95 °C for 5 min; 20 cycles of denaturation at 95 °C for 30 s, annealing at 50 °C for 30 s, and extension at 72 °C for 40 s; followed by a final extension at 72 °C for 7 min.

PCR products were purified using the Omega DNA Purification Kit (Omega Bio-tek, Norcross, GA, USA) and quantified using a Qsep-400 system (BiOptic Inc., New Taipei City, Taiwan). Purified amplicons were pooled to construct the sequencing library and subjected to paired-end sequencing (2 × 250 bp) on an Illumina NovaSeq 6000 platform (Beijing Biomarker Technologies Co., Ltd., Beijing, China).

Initial sequence processing was conducted using the BMKCloud bioinformatic platform. Raw paired-end reads were quality-filtered using Trimmomatic version 0.33. A 50-bp sliding window was applied, and sequences were truncated when the mean quality score within the window was below 20. Primer sequences were identified and removed using Cutadapt version 1.9.1, allowing a maximum mismatch rate of 20% and requiring a minimum primer coverage of 80%.

Paired-end reads were merged using USEARCH version 10, with a minimum overlap length of 10 bp, a minimum similarity of 90% within the overlapping region, and a maximum of five mismatched bases. Chimeric sequences were identified and removed using UCHIME version 8.1.

Amplicon sequence variants were inferred from the quality-controlled sequences using the DADA2 method within QIIME 2 version 2020.6.0 (Bolyen et al., 2019; Callahan et al., 2016). Features with fewer than two reads across all samples were removed. Taxonomic annotation was performed using a Naive Bayes classifier implemented in QIIME 2 against the SILVA release 138 reference database, with a confidence threshold of 0.70 (Quast et al., 2013). Taxonomic nomenclature throughout the manuscript, figures, tables, and **Supplementary Material** followed the SILVA release 138 annotation framework used in the original bioinformatic analysis. Microbial composition was summarized at the phylum, family, and genus levels, with genus-level data used for the primary differential-abundance analysis.

Alpha diversity was evaluated using the ACE, Chao1, Shannon, and Simpson indices. Between-group comparisons were performed using two-sided Wilcoxon rank-sum tests in R version 4.6.0. Beta diversity was evaluated using principal coordinates analysis based on Bray–Curtis distances. Between-group differences in overall microbial community composition were assessed using the adonis2 function in the R package vegan version 2.7.5, with 9,999 permutations. Homogeneity of multivariate dispersion was additionally evaluated using the betadisper and permutest functions in vegan, also with 9,999 permutations.

For the primary genus-level differential-abundance analysis, raw genus-level count data were analyzed using ANCOM-BC2, as implemented in the ANCOMBC package version 2.14.0 in R version 4.6.0 (Lin and Peddada, 2024). Genera detected in at least 20% of the 26 samples, corresponding to detection in at least six samples, were retained. Study group was included as the fixed effect, and the HC group was used as the reference group. P values were adjusted using the Benjamini–Hochberg procedure. Pseudo-count sensitivity analysis was enabled because of the presence of zero counts, and structural zeros were evaluated separately. A genus was considered robustly differentially abundant only when the FDR-adjusted q value was below 0.05 and the result passed the pseudo-count sensitivity analysis. Bias-corrected log fold changes, standard errors, 95% confidence intervals, raw P values, and FDR-adjusted q values were reported.

Sample-level heatmaps and boxplots were generated from relative-abundance data for descriptive visualization and were not treated as independent confirmatory statistical tests. The original STAMP-based relative-abundance comparison was retained only as a secondary exploratory analysis in the **Supplementary Material**. Taxa identified solely using raw P values in the secondary analysis were not interpreted as FDR-confirmed differential taxa. All microbiome figures presented in the final manuscript were generated or redrawn in R version 4.6.0 using the processed sequencing data.

The same quality-control, diversity, and differential-abundance procedures were applied in the prespecified infection-confirmation and infection-site sensitivity analyses described in Section 2.7.

The sequencing provider generated PICRUSt2-based inferred functional profiles as part of its standard bioinformatic deliverables. However, these outputs were not included in the analytical framework, statistical analyses, figures, tables, or biological interpretation of the present manuscript. All pathway-enrichment findings reported in this study were derived exclusively from metabolic features directly measured using LC–MS/MS-based untargeted fecal metabolomics.

### 2.4 Untargeted fecal metabolomic profiling

Untargeted fecal metabolomic profiling was performed using an LC–MS/MS platform. A total of 26 fecal samples from the HC and SAI groups were included. Overall, 2,956 metabolic features were detected, including 1,520 features in positive-ion mode and 1,436 features in negative-ion mode; 2,491 features received putative MS/MS-based annotations.

Fecal samples stored at −80 °C were thawed on ice. Approximately 20 mg of each fecal sample was transferred to a centrifuge tube, and 400 μL of 70% methanol–water extraction solution containing internal standards was added. Samples were vortexed for 3 min, sonicated in an ice-water bath for 10 min, vortexed for an additional 1 min, and incubated at −20 °C for 30 min. The samples were centrifuged at 12,000 rpm for 10 min at 4 °C, and 300 μL of the supernatant was transferred to a new tube. After a second centrifugation at 12,000 rpm for 3 min at 4 °C, 200 μL of supernatant was transferred to an autosampler vial for LC–MS/MS analysis.

Chromatographic separation was performed using a Waters ACQUITY Premier HSS T3 column (1.8 μm, 2.1 × 100 mm) on a Thermo Scientific Vanquish ultrahigh-performance liquid chromatography system. Mobile phase A consisted of 0.1% formic acid in water, and mobile phase B consisted of 0.1% formic acid in acetonitrile. The gradient was programmed as follows: 5% B at 0 min, 20% B at 1 min, 99% B at 3 min, maintained at 99% B until 4.5 min, returned to 5% B at 4.6 min, and maintained at 5% B until 6 min. The column temperature was 40 °C, the flow rate was 0.4 mL/min, and the injection volume was 3 μL.

Mass-spectrometric detection was performed using a Q Exactive HF-X instrument in positive- and negative-electrospray-ionization modes. The spray voltages were 3.8 kV in positive mode and 3.4 kV in negative mode. The sheath-gas and auxiliary-gas settings were 60 and 20 arbitrary units, respectively, and the ion-transfer-tube temperature was 320 °C. MS1 spectra were acquired over an m/z range of 70–1,000 at a resolution of 60,000 with an automatic-gain-control target of 3.0 × 10^6^. MS2 spectra were acquired over an m/z range of 70–1,000 at a resolution of 15,000 with an automatic-gain-control target of 1.0 × 10^5^. Stepped collision energies of 30, 40, and 50 V were used.

Raw mass-spectrometry files were converted to mzML format using ProteoWizard and processed using XCMS for peak extraction, peak alignment, and retention-time correction within the vendor-provided untargeted metabolomic workflow. The archived project report did not retain the individual ProteoWizard or XCMS component-version numbers.

Features with missing values in more than 50% of samples within a study group were removed. Remaining missing values were imputed using a combination of K-nearest-neighbor imputation and one-fifth of the minimum nonzero value according to the vendor’s preprocessing workflow. Support-vector-regression-based signal correction was applied to reduce systematic analytical drift.

Metabolic features were annotated by matching experimental mass and MS/MS information against the vendor’s in-house spectral library, integrated public databases, and prediction libraries. Features with an integrated annotation score above 0.5 and a coefficient of variation below 0.30 in quality-control samples were retained. Positive- and negative-ion-mode results were subsequently merged. When duplicate annotations were present, the feature with the highest annotation confidence and integrated annotation score was retained.

Metabolite identities were classified according to the available annotation evidence. Features confirmed using an authentic reference standard with concordant retention time and MS/MS fragmentation would be regarded as level-1 identifications. Features assigned using MS/MS spectral-library matching without authentic-standard confirmation were regarded as putatively annotated compounds, whereas features supported only at the chemical-class or formula level were regarded as putatively characterized compound classes. Because authentic-standard confirmation was not systematically performed in the present untargeted analysis, the reported metabolite names were treated as putative annotations rather than definitive level-1 identifications.

### 2.5 Screening of candidate metabolic features and metabolomics-based pathway-enrichment analysis

After preprocessing and normalization, unsupervised principal component analysis was performed using the base prcomp function in R version 4.1.2 after log_2_ transformation and zero-centering to describe the global distribution of fecal metabolic profiles. The final PCA plot was generated in R version 4.6.0 using the processed metabolic-feature matrix.

The original exploratory OPLS-DA model and the corresponding 200 random-label permutations were generated by the metabolomics service provider using MetaboAnalystR version 1.0.1 in R version 4.1.2 within the vendor’s metabonomics pipeline version 6.7, after log_2_ transformation and unit-variance scaling (Chong et al., 2019). The model was summarized using cumulative R^2^X, cumulative R^2^Y, and cumulative Q^2^ values.

The final OPLS-DA score plot was redrawn in R version 4.6.0 using the archived numerical score coordinates from the original vendor-generated model. The archived permutation-validation output was retained because the individual numerical results for all 200 permuted models were not available for independent reconstruction. The OPLS-DA model was not refitted using the ropls package. Because OPLS-DA is supervised and the study included a limited number of samples, the OPLS-DA result was not interpreted as independent confirmatory evidence of group discrimination and was presented only in **Supplementary Figure 1**.

All other metabolomic plots presented in the final manuscript, including the volcano plot, clustered heatmap, and pathway-enrichment plots, were generated or redrawn in R version 4.6.0 using processed data or archived analytical results.

Candidate metabolic features were screened by integrating multivariate and univariate analyses using VIP > 1 and raw P < 0.05 as the predefined exploratory criteria. Benjamini–Hochberg FDR-adjusted q values were also calculated and reported. Features that did not remain significant after FDR correction were interpreted as exploratory candidate metabolic features rather than validated differential metabolites or biomarkers.

Candidate metabolic features were annotated using the KEGG Compound, KEGG Pathway, and Human Metabolome Database resources. KEGG pathway enrichment, HMDB annotation, and metabolite set enrichment analysis were performed using the vendor-provided metabolomic annotation outputs and were visualized in R. These analyses were based exclusively on metabolic features directly measured using LC–MS/MS-based untargeted fecal metabolomics. They did not involve PICRUSt2-derived results, predicted microbial gene content, or other 16S rRNA-based functional inference.

Because candidate features were selected using VIP > 1 and raw P < 0.05, pathway-enrichment findings based on unadjusted P values were treated as exploratory and hypothesis-generating and were not regarded as FDR-confirmed pathway alterations.

### 2.6 Integrated microbiome–metabolome analysis

To explore potential links among gut microbial alterations, fecal metabolic profiles, and clinical phenotypes, the four genus-level taxa meeting the predefined robust ANCOM-BC2 criterion were integrated with candidate metabolic features and clinical indicators from patients with SAI. Spearman rank correlation analysis was used because several clinical and omics variables showed skewed distributions.

Before correlation analysis, all clinical variables were checked against the source records for impossible values, transcription errors, unit inconsistencies, and potentially extreme observations. Continuous clinical variables were screened using the Q1 ± 3 × IQR rule. Binary and categorical variables were not subjected to IQR-based outlier screening. Values flagged by this procedure were manually verified against the source records, and no genuine clinical observation was excluded solely because it was statistically extreme.

Initial exploratory associations were defined using |rho| > 0.5 and raw P < 0.05. To assess whether individual observations disproportionately influenced the correlation estimates, pair-specific leave-one-out sensitivity analyses were performed. For each association, the correlation was recalculated after sequential omission of each complete observation.

An association was considered sensitivity-supported when the full-data analysis met |rho| > 0.5 and raw P < 0.05, the correlation direction remained unchanged across all valid leave-one-out iterations, and at least 80% of the valid iterations continued to meet |rho| > 0.5 and raw P < 0.05. Only sensitivity-supported associations were retained in the revised correlation networks.

Benjamini–Hochberg FDR-adjusted q values were also calculated and reported. Because of the limited sample size and the large number of pairwise comparisons, all correlation analyses were regarded as exploratory and hypothesis-generating and were not interpreted as independent, predictive, mechanistic, or causal evidence.

### 2.7 Statistical analysis

Revised statistical analyses and final figure generation were performed in R version 4.6.0. The original vendor-generated metabolomic multivariate analyses were performed in R version 4.1.2 within the vendor’s metabonomics pipeline version 6.7. IBM SPSS Statistics was not used for the analyses reported in the final revised manuscript.

Continuous variables were summarized as mean ± standard deviation or median and interquartile range according to their distributions, whereas categorical variables were summarized as counts and percentages. Age was compared between the HC and SAI groups using a two-sided Mann–Whitney U test, and sex distribution was compared using Fisher’s exact test.

Microbial alpha-diversity indices were compared between groups using two-sided Wilcoxon rank-sum tests. P values were calculated using the asymptotic normal approximation with continuity correction. Bray–Curtis beta-diversity differences were assessed using the adonis2 function in the vegan package version 2.7.5 with 9,999 permutations. Homogeneity of multivariate dispersion was evaluated using the betadisper and permutest functions in vegan, also with 9,999 permutations.

Primary genus-level differential-abundance analysis was performed using ANCOM-BC2, implemented in the ANCOMBC package version 2.14.0. Genera detected in at least 20% of the 26 samples were retained. Study group was included as the fixed effect, and the HC group was used as the reference group. P values were adjusted using the Benjamini–Hochberg procedure. A genus was considered robustly differentially abundant only when the FDR-adjusted q value was below 0.05 and the result passed the pseudo-count sensitivity analysis.

Two prespecified exploratory sensitivity analyses were performed. First, alpha-diversity, Bray–Curtis beta-diversity, and ANCOM-BC2 analyses were repeated after excluding the four patients whose respiratory infection was confirmed using bronchoalveolar lavage fluid next-generation sequencing, thereby restricting the SAI group to 12 culture-confirmed cases. Second, these analyses were repeated after excluding the two patients with bloodstream infection, thereby restricting the SAI group to 14 patients with respiratory tract infection. The same prevalence threshold, FDR correction, structural-zero assessment, and pseudo-count sensitivity settings were applied to each restricted dataset. Because of the reduced sample sizes, interpretation focused on effect direction, effect magnitude, confidence intervals, FDR-adjusted q values, and pseudo-count sensitivity rather than requiring every association to retain robust significance.

As a limited antimicrobial-exposure sensitivity analysis, alpha-diversity comparisons were repeated after excluding the only patient with SAI who had not received antimicrobial treatment before fecal sampling. This analysis was not regarded as controlling for antimicrobial confounding because all remaining patients with SAI were antimicrobial-exposed before sampling, whereas none of the healthy controls had received antibiotics before sampling.

Metabolomic PCA was performed using the base prcomp function in R. Candidate metabolic features were screened using VIP > 1 and raw P < 0.05 and were interpreted as exploratory unless they remained significant after FDR correction. The exploratory OPLS-DA model was summarized using cumulative R^2^X, cumulative R^2^Y, and cumulative Q^2^ values and was evaluated using 200 random-label permutations.

Spearman rank correlations and Benjamini–Hochberg-adjusted q values were calculated in R. Pair-specific leave-one-out sensitivity analyses were implemented using custom R scripts. Data manipulation and reshaping were performed using dplyr version 1.2.1 and tidyr version 1.3.2. Statistical figures and heatmaps were generated using ggplot2 version 4.0.3 and pheatmap version 1.0.13 and were assembled using patchwork version 1.3.2. All statistical tests were two-sided.

### 2.8 Ethics statement

This study was conducted in accordance with the Declaration of Helsinki and was approved by the Ethics Committee of the Sixth Medical Center of Chinese PLA General Hospital, formerly the Navy General Hospital (approval No. HZKY-PJ-2024-65; approval date: January 3, 2025). The study was conducted under the approved project entitled “Mechanisms of vascular endothelial injury and therapeutic strategies in severe infection caused by biological injury.” Written informed consent was obtained from all participants or their legal representatives before biospecimen collection and clinical data acquisition. All participant information was de-identified before analysis, and confidentiality was maintained throughout the study.

## 3 Results

### 3.1 Study population, clinical characteristics, and multi-omics workflow

A total of 26 fecal samples were included, comprising 10 samples from HCs and 16 samples from patients with SAI. All fecal samples underwent both 16S rRNA sequencing and untargeted fecal metabolomic profiling. Age and sex data were available for all participants, whereas disease-related laboratory indicators, severity scores, organ-support measures, complications, and clinical outcomes were available only for patients with SAI.

The median age was 75.00 (71.75–80.25) years in the HC group and 72.50 (51.75–77.75) years in the SAI group, with no statistically significant between-group difference (P = 0.460). The HC group included five men (50.0%), compared with 10 men (62.5%) in the SAI group, and sex distribution did not differ significantly between the groups (P = 0.689) (**Table 1**).

**Table 1:** Baseline demographic characteristics of the HC and SAI groups and clinical characteristics of patients with SAI.

Characteristic	HC, n = 10	SAI, n = 16	P value
Demographics			
Age, years	75.00 (71.75–80.25)	72.50 (51.75–77.75)	0.460
Male sex	5 (50.0)	10 (62.5)	0.689
Patient source			
Respiratory general ward	—	1 (6.3)	—
Other general ward	—	8 (50.0)	—
Respiratory intensive care unit	—	5 (31.3)	—
Other intensive care unit	—	2 (12.5)	—
Infection and microbiological characteristics			
Respiratory tract infection	—	14 (87.5)	—
Bloodstream infection	—	2 (12.5)	—
PICC-related bloodstream infection	—	1 (6.3)	—
Sputum culture confirmation	—	10 (62.5)	—
BALF NGS confirmation	—	4 (25.0)	—
Blood culture confirmation	—	2 (12.5)	—
Treatment exposure before fecal sampling			
Antimicrobial exposure before fecal sampling	0 (0.0)	15 (93.8)	—
No antimicrobial exposure before fecal sampling	10 (100.0)	1 (6.3)	—
Inflammatory and laboratory indicators			
White blood cell count, ×10^9^/L	—	9.38 (5.71–14.94)	—
Neutrophil count, ×10^9^/L	—	6.57 (4.25–10.50)	—
C-reactive protein, mg/L	—	19.35 (10.38–86.80)	—
Procalcitonin, ng/mL	—	0.50 (0.38–1.12)	—
Creatinine, μmol/L	—	60.90 (56.33–76.98)	—
Albumin, g/L	—	36.05 (32.58–38.75)	—
Lactate, mmol/L	—	1.50 (1.07–1.85)	—
Disease severity			
APACHE II score	—	15.50 (12.75–18.50)	—
SOFA score	—	5.00 (3.50–5.25)	—
Complications and organ support			
ARDS	—	5 (31.3)	—
Sepsis without septic shock	—	2 (12.5)	—
Septic shock	—	3 (18.8)	—
Total sepsis	—	5 (31.3)	—
CRRT	—	2 (12.5)	—
Circulatory failure	—	4 (25.0)	—
SBP <90 mmHg after fluid resuscitation	—	1/15 (6.7)	—
Vasopressor use after fluid resuscitation	—	2 (12.5)	—
Clinical outcomes			
Improved discharge	—	13 (81.3)	—
Discharge against medical advice	—	1 (6.3)	—
Death	—	2/15 (13.3)	—

For patients with SAI, the first available qualified fecal sample was collected within 7 days after microbiological confirmation of *S. aureus* infection. The date and time of fecal collection were recorded. Laboratory and disease-severity variables used in the clinical–omics analysis were obtained within 24 h of fecal collection, with the temporally closest measurement selected when more than one value was available.

Among the 16 patients with SAI, 14 patients (87.5%) had respiratory tract infections and two patients (12.5%) had bloodstream infections, including one documented PICC-related bloodstream infection. Microbiological confirmation was based on sputum culture in 10 patients (62.5%), detection of *S. aureus* in bronchoalveolar lavage fluid by next-generation sequencing in four patients (25.0%), and blood culture in two patients (12.5%). All 16 SAI cases met the predefined clinical adjudication criteria for active infection. For respiratory tract cases, microbiological evidence was interpreted together with compatible respiratory manifestations, inflammatory indicators, and imaging findings. No case classified as probable airway colonization or specimen contamination was retained in the final cohort.

Fifteen of the 16 patients with SAI (93.8%) had received antimicrobial treatment before fecal sample collection, whereas one patient (6.3%) had not received antimicrobial treatment before sampling. None of the healthy controls had received antibiotics before fecal collection. Patient-level infection characteristics and pre-sampling antimicrobial agents are provided in **Supplementary Table 1**. Although the temporal ordering of antimicrobial exposure and fecal sampling was confirmed, the exact treatment-to-sampling interval, treatment duration, dosage, and cumulative pre-sampling antimicrobial exposure could not be consistently quantified.

**Figure 1:**
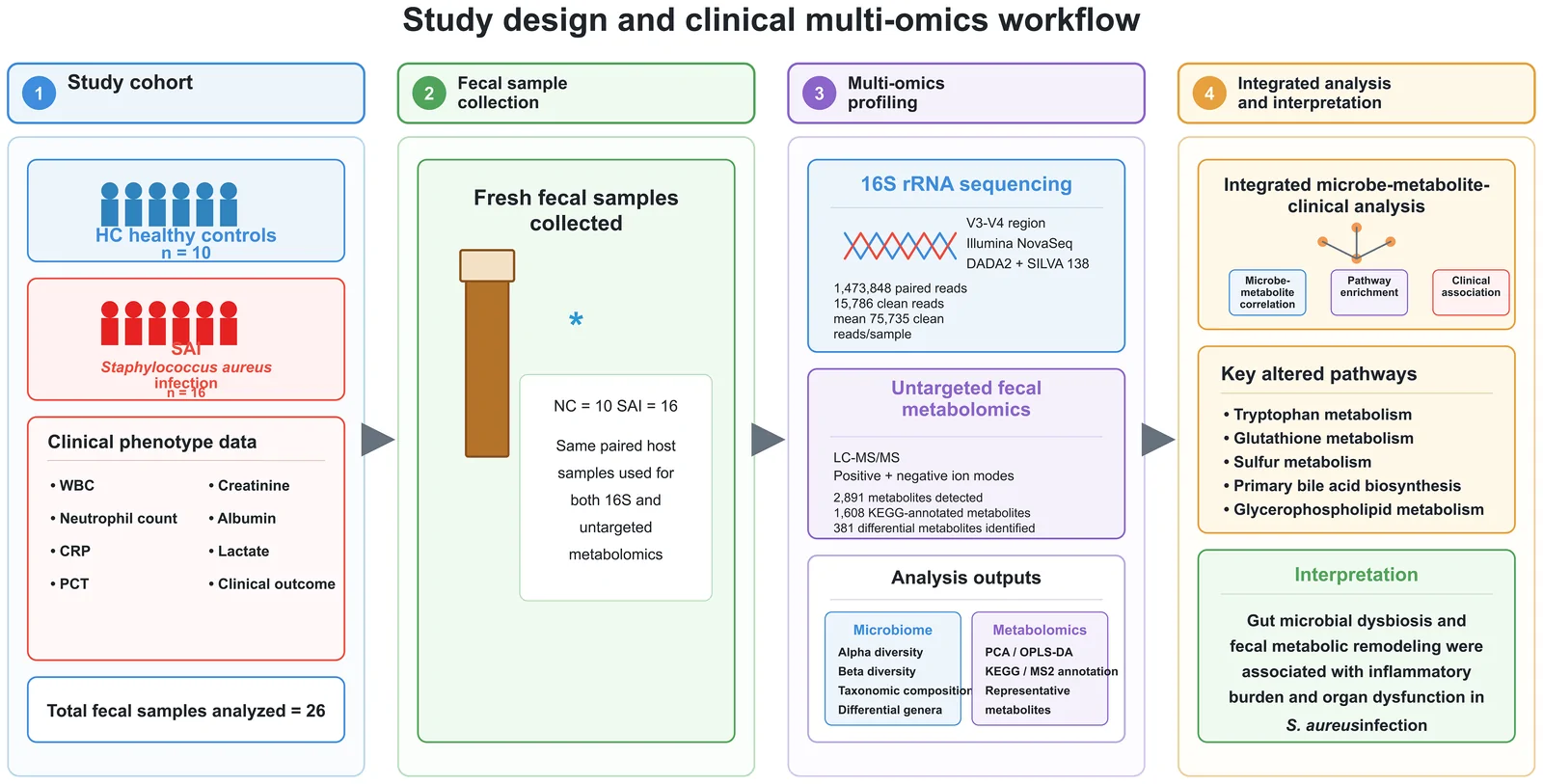
Study design and clinical multi-omics workflow. HC, healthy control; SAI, *Staphylococcus aureus* infection; CRP, C-reactive protein; PCT, procalcitonin; APACHE II, Acute Physiology and Chronic Health Evaluation II; SOFA, Sequential Organ Failure Assessment; ARDS, acute respiratory distress syndrome; CRRT, continuous renal replacement therapy; SBP, systolic blood pressure.

In the SAI group, the white blood cell count was 9.38 (5.71–14.94) × 10^9^/L, the neutrophil count was 6.57 (4.25–10.50) × 10^9^/L, the CRP concentration was 19.35 (10.38–86.80) mg/L, the PCT concentration was 0.50 (0.38–1.12) ng/mL, the creatinine concentration was 60.90 (56.33–76.98) μmol/L, the albumin concentration was 36.05 (32.58–38.75) g/L, and the lactate concentration was 1.50 (1.07–1.85) mmol/L. Thirteen patients (81.3%) were discharged after clinical improvement (**Table 1**). The analytical workflow included fecal sample collection, 16S rRNA sequencing, untargeted fecal metabolomics, genus-level differential-abundance analysis, candidate metabolic-feature screening, metabolomics-based pathway-enrichment analysis, microbiome–metabolome correlation analysis, and clinical–omics correlation analysis (**Figure 1**).

Continuous variables are presented as median (interquartile range), and categorical variables are presented as n (%). Age was compared between the HC and SAI groups using a two-sided Mann–Whitney U test, and sex distribution was compared using Fisher’s exact test. Disease-related laboratory indicators, severity scores, organ-support measures, complications, and clinical outcomes were available only for patients with SAI and were therefore not compared between groups. Pre-sampling antimicrobial exposure was determined from the prospectively collected medication records. Fifteen patients with SAI had received antimicrobial treatment before fecal sampling, whereas one patient had not. None of the healthy controls had received antibiotics before sampling. The exact treatment-to-sampling interval, treatment duration, dose, and cumulative pre-sampling exposure could not be consistently quantified. Dashes indicate that the corresponding variable was not collected for the HC group or that a between-group comparison was not applicable. PCT was missing in one patient, lactate was missing in five patients, systolic blood pressure after fluid resuscitation was missing in one patient, and mortality status was missing in one patient.

This study included the HC and SAI groups. Fresh fecal samples were collected, and paired 16S rRNA sequencing and untargeted fecal metabolomics were performed using the same samples. The 16S rRNA sequencing workflow included alpha-diversity analysis, Bray–Curtis beta-diversity analysis, taxonomic-composition analysis, and genus-level differential-abundance analysis using ANCOM-BC2. Untargeted metabolomics was used to characterize global metabolic profiles, screen candidate metabolic features, and perform exploratory metabolomics-based pathway-enrichment analysis. Robust genus-level taxa identified using ANCOM-BC2, candidate metabolic features, and clinical indicators from patients with SAI were subsequently integrated in exploratory microbiome–metabolome and clinical–omics correlation analyses. Vendor-generated PICRUSt2-based inferred functional profiles were not included in the analytical framework, figures, or biological interpretation of the present manuscript. HC, healthy control; SAI, *Staphylococcus aureus* infection; LC–MS/MS, liquid chromatography–tandem mass spectrometry; CRP, C-reactive protein; PCT, procalcitonin.

### 3.2 Patients with SAI exhibit reduced gut microbial alpha diversity and modest group-associated differences in overall community structure

Across the 26 fecal samples, 16S rRNA sequencing generated 1,879,844 paired raw reads. After paired-end read quality control and merging, 1,734,540 clean reads were obtained. Each sample yielded at least 47,944 clean reads, with an average of 66,713 clean reads per sample. DADA2 denoising generated 9,538 features, corresponding to 1,451,069 sequences.

**Figure 2:**
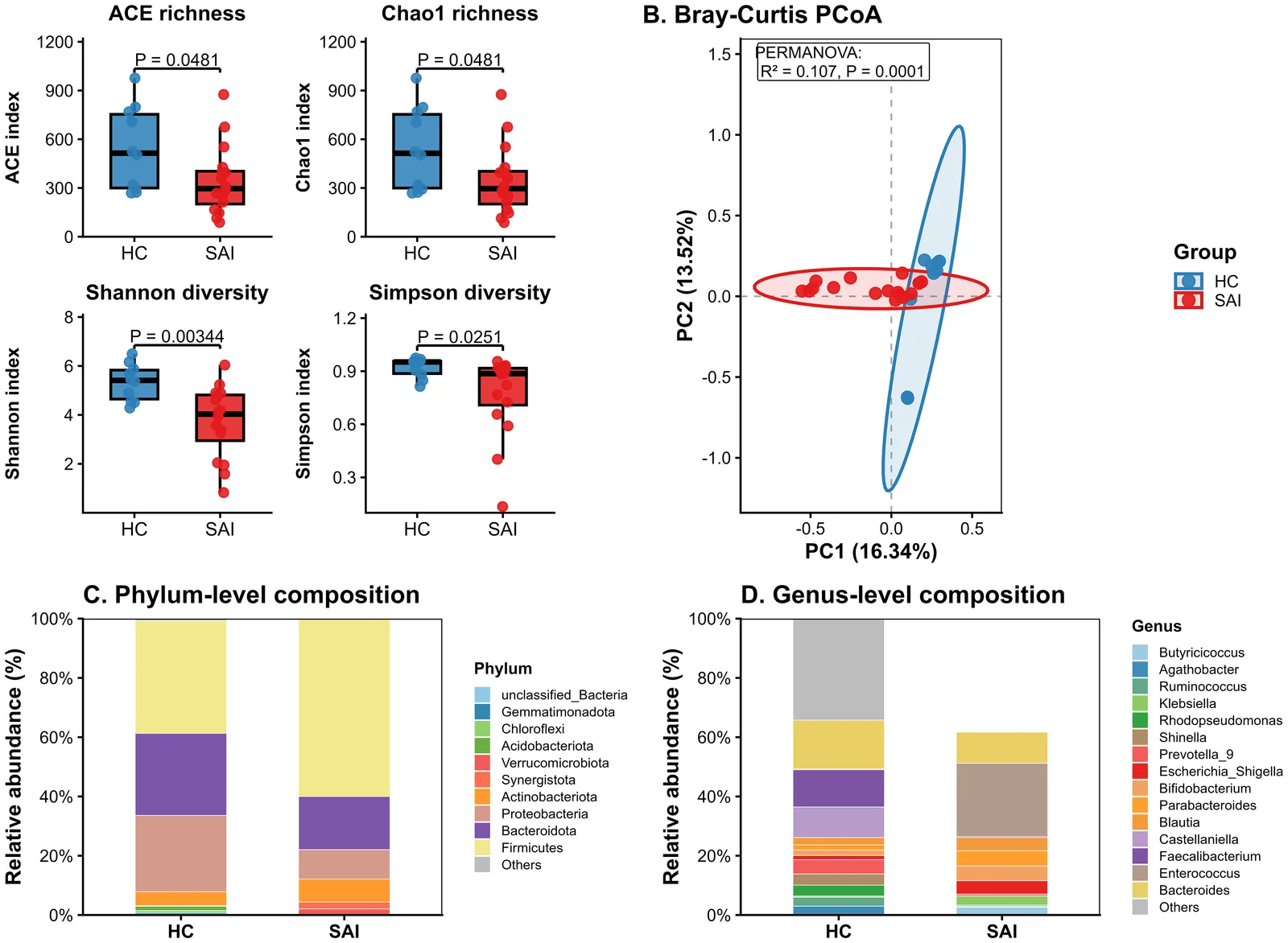
Gut microbial diversity and taxonomic composition in the HC and SAI groups. **(A)** Comparisons of ACE richness, Chao1 richness, Shannon diversity, and Simpson diversity between the HC and SAI groups. Boxplots show medians and interquartile ranges, and points represent individual samples. Between-group comparisons of alpha-diversity indices were performed using two-sided Wilcoxon rank-sum tests. ACE and Chao1 yielded identical test statistics and P values (W = 118 and P = 0.0481 for both indices). **(B)** Principal coordinates analysis based on Bray–Curtis distances. Each point represents one sample, and ellipses summarize the distribution of samples within each group. PC1 and PC2 explained 16.34% and 13.52% of the total variation, respectively.PERMDISP did not indicate a statistically significant difference in multivariate dispersion between groups (F = 3.2631, permutation P = 0.0885). Substantial sample overlap remained, and the ordination was therefore interpreted as showing modest group-associated differentiation rather than clear separation. **(C)** Stacked bar plot of the mean relative abundance of major bacterial phyla. **(D)** Stacked bar plot of the mean relative abundance of major bacterial genera. HC, healthy control; SAI, *Staphylococcus aureus* infection; PCoA, principal coordinates analysis; PERMANOVA, permutational multivariate analysis of variance; PERMDISP, permutational analysis of multivariate dispersions.

Alpha-diversity analysis showed that ACE and Chao1 richness were both lower in the SAI group than in the HC group. The two comparisons yielded identical Wilcoxon rank-sum statistics and P values because the ACE and Chao1 measurements produced the same between-group rank ordering (ACE, W = 118, P = 0.0481; Chao1, W = 118, P = 0.0481). Shannon diversity was also lower in the SAI group than in the HC group (W = 136, P = 0.00344), and Simpson diversity differed between groups (W = 123, P = 0.0251) (**Figure 2A**). These findings indicate that patients with SAI had reduced gut microbial richness and diversity compared with HCs.

As a limited sensitivity analysis, alpha-diversity comparisons were repeated after excluding the only patient who had not received antimicrobial treatment before fecal sampling. Shannon and Simpson diversity remained significantly different between groups (P = 0.0051 and P = 0.0375, respectively), whereas the differences in ACE and Chao1 richness became borderline (both P = 0.0557). This analysis did not control for antimicrobial confounding because all 15 remaining patients with SAI had received antimicrobial treatment before fecal sampling, whereas none of the healthy controls had received antibiotics before sampling.

Bray–Curtis PCoA showed substantial overlap between the HC and SAI samples, together with a modest trend toward group-associated differentiation. PC1 and PC2 explained 16.34% and 13.52% of the total variation, respectively. PERMANOVA indicated a statistically significant but modest group-associated difference in overall microbial community composition, with study group explaining approximately 10.7% of the total variation (F = 2.8654, R^2^ = 0.1067, P = 0.0001). PERMDISP did not indicate a statistically significant difference in multivariate dispersion between the HC and SAI groups (F = 3.2631, permutation P = 0.0885). Nevertheless, because the PERMANOVA effect size was modest and substantial sample overlap remained, the ordination result was interpreted as modest group-associated differentiation rather than clear separation (**Figure 2B**).At the taxonomic composition level, the gut microbiota of both groups was mainly composed of Firmicutes, Bacteroidota, Proteobacteria, and Actinobacteriota. Phylum- and genus-level stacked bar plots indicated differences in the relative abundance of major taxa between the HC and SAI groups (**Figures 2C, D**).

### 3.3 ANCOM-BC2 identifies four robust genus-level differences between the HC and SAI groups

Raw genus-level count data were analyzed using ANCOM-BC2. Of the 643 genera retained after initial data cleaning, 163 were detected in at least 20% of the 26 samples and were included in the differential-abundance analysis. After Benjamini–Hochberg correction and pseudo-count sensitivity analysis, four genera met the predefined robust-significance criterion.

**Figure 3:**
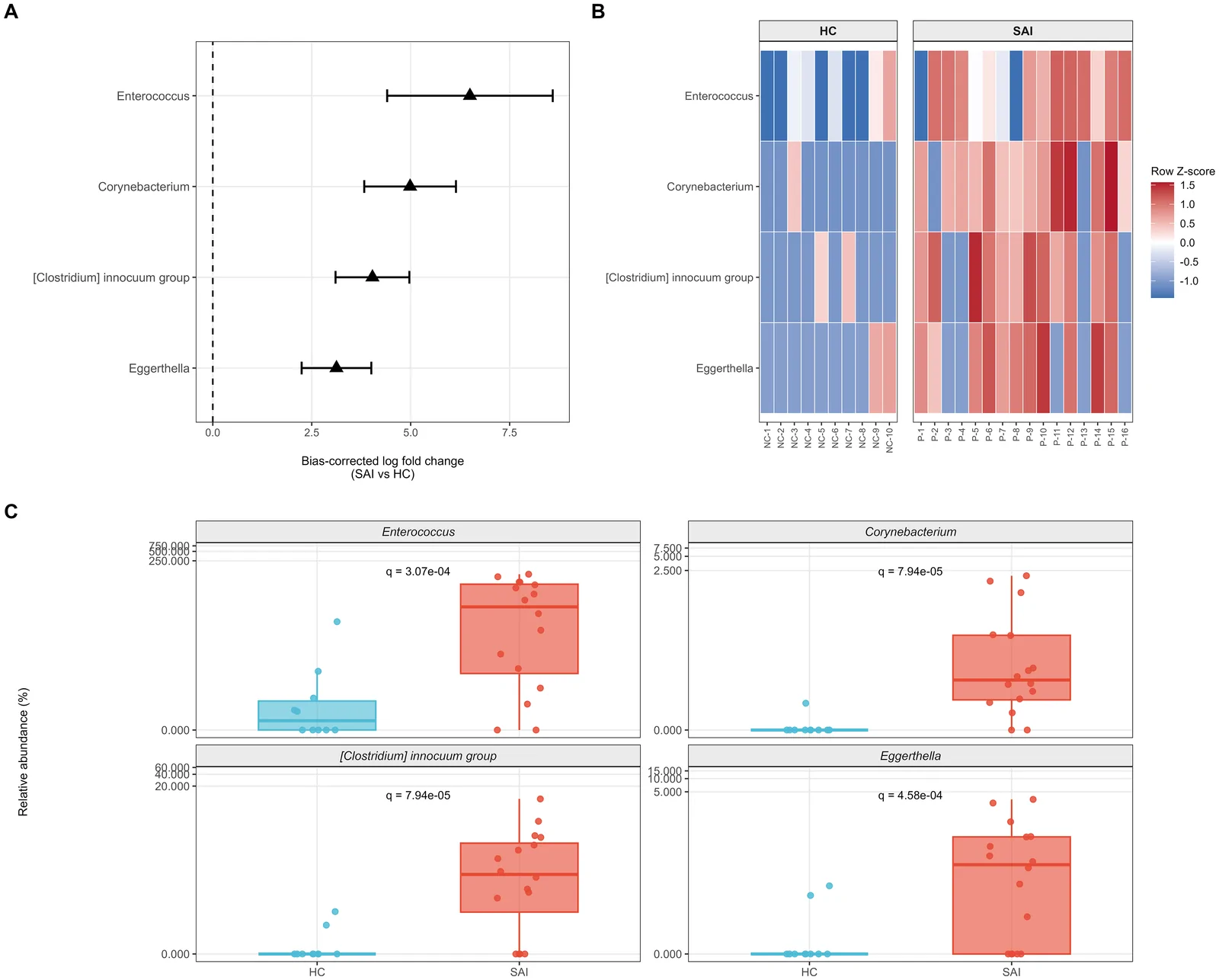
Genus-level microbial patterns identified using compositionally aware differential-abundance analysis. **(A)** Bias-corrected effect estimates for robustly differentially abundant genera identified using ANCOM-BC2. Points represent bias-corrected log fold changes for the SAI group relative to the HC group, and error bars represent 95% confidence intervals. Only genera with an FDR-adjusted q value < 0.05 that passed the pseudo-count sensitivity analysis are displayed. Positive values indicate higher bias-corrected abundance in the SAI group. **(B)** Sample-level heatmap of the four robustly differentially abundant genera. Values represent row-wise Z-scores calculated from log10-transformed relative abundances. **(C)** Relative-abundance distributions of the four robust genera in the HC and SAI groups. Boxes represent the interquartile range, horizontal lines represent the median, and points represent individual samples. The displayed q values were obtained from the ANCOM-BC2 analysis. Panels B and C are descriptive visualizations of relative-abundance patterns and should not be interpreted as independent confirmatory statistical tests. HC, healthy control; SAI, *Staphylococcus aureus* infection; CI, confidence interval; FDR, false discovery rate.

Compared with the HC group, *Corynebacterium*, *[Clostridium] innocuum* group, *Enterococcus*, and *Eggerthella* showed higher bias-corrected abundance in the SAI group. The corresponding bias-corrected log fold changes were 4.986, 4.034, 6.498, and 3.125, respectively. The 95% confidence intervals were 3.827–6.145 for *Corynebacterium*, 3.101–4.967 for *[Clostridium] innocuum* group, 4.406–8.590 for *Enterococcus*, and 2.245–4.006 for *Eggerthella*. Their FDR-adjusted q values ranged from 7.94 × 10^−5^ to 4.58 × 10^−4^, and all four results passed the pseudo-count sensitivity analysis (**Figure 3A**; **Table 2**). No genus showing lower bias-corrected abundance in the SAI group met the predefined robust-significance criterion.

**Table 2:** Robust genus-level differential abundance identified using ANCOM-BC2.

Genus	Bias-corrected log fold change	95% CI	Raw P value	FDR-adjusted q value	Direction
*Corynebacterium*	4.986	3.827–6.145	1.25 × 10^−6^	7.94 × 10^−5^	Higher in SAI
*[Clostridium] innocuum* group	4.034	3.101–4.967	2.08 × 10^−6^	7.94 × 10^−5^	Higher in SAI
*Enterococcus*	6.498	4.406–8.590	1.21 × 10^−5^	3.07 × 10^−4^	Higher in SAI
*Eggerthella*	3.125	2.245–4.006	2.40 × 10^−5^	4.58 × 10^−4^	Higher in SAI

Sensitivity analyses were conducted after restricting the SAI group to the 12 culture-confirmed cases and, separately, to the 14 patients with respiratory tract infection. In the culture-confirmed subset, the between-group differences in Shannon and Simpson diversity remained statistically significant (P = 0.0111 and P = 0.0321, respectively), whereas the ACE and Chao1 richness differences were attenuated (both P = 0.1563). In the respiratory-infection-only analysis, the differences in ACE, Chao1, Shannon, and Simpson indices remained statistically significant (P = 0.0326, P = 0.0326, P = 0.0017, and P = 0.0128, respectively).

Bray–Curtis PERMANOVA remained statistically significant in both restricted analyses. In the culture-confirmed subset, study group explained 11.3% of the variation in community composition (F = 2.547, R^2^ = 0.113, P = 0.0004). However, PERMDISP was also statistically significant (P = 0.0372), indicating that the corresponding PERMANOVA result may have been influenced partly by unequal within-group dispersion. In the respiratory-infection-only analysis, study group explained 11.8% of the variation (F = 2.956, R^2^ = 0.118, P = 0.0001), whereas PERMDISP was not statistically significant (P = 0.2268).

The bias-corrected effect estimates for *Corynebacterium*, the *[Clostridium] innocuum* group, *Enterococcus*, and *Eggerthella* remained positive in both restricted analyses. In the culture-confirmed subset, the corresponding bias-corrected log fold changes were 5.373, 4.075, 5.652, and 3.462, respectively. *Corynebacterium* and the *[Clostridium] innocuum* group retained pseudo-count-robust significance, whereas the estimates for *Enterococcus* and *Eggerthella*, although FDR-significant, did not pass the pseudo-count sensitivity criterion.

In the respiratory-infection-only analysis, the corresponding bias-corrected log fold changes were 4.918, 3.718, 6.749, and 2.830, respectively. *Corynebacterium*, the *[Clostridium] innocuum* group, and *Enterococcus* retained pseudo-count-robust significance. The *Eggerthella* estimate remained FDR-significant but did not pass the pseudo-count sensitivity criterion. Complete results are provided in **Supplementary Table 1**, sheet S9_Infection_Sensitivity, and **Supplementary Figure 2**.

The sample-level heatmap and boxplots illustrated the relative-abundance distributions of these four genera across the HC and SAI samples (**Figures 3B, C**). These panels were used for descriptive visualization, whereas statistical inference was based on ANCOM-BC2. Additional taxa identified in the original STAMP-based relative-abundance analysis are reported only as secondary exploratory findings in **Supplementary Table 1**, sheet S3b_Secondary_STAMP, and are not interpreted as robust differential taxa.

Genus-level differential abundance was analyzed using ANCOM-BC2. Genera detected in at least 20% of the 26 samples were retained. Study group was included as the fixed effect, and the HC group was used as the reference group. Positive bias-corrected log fold changes indicate higher abundance in the SAI group. P values were adjusted using the Benjamini–Hochberg procedure. Only genera with an FDR-adjusted q value < 0.05 that passed the pseudo-count sensitivity analysis are shown. HC, healthy control; SAI, *Staphylococcus aureus* infection; CI, confidence interval; FDR, false discovery rate. Results of the culture-confirmed and respiratory-infection-only sensitivity analyses are provided in **Supplementary Table 1**, sheet S9_Infection_Sensitivity.

### 3.4 Fecal metabolic profiles show modest group-associated differences between the HC and SAI groups

**Figure 4:**
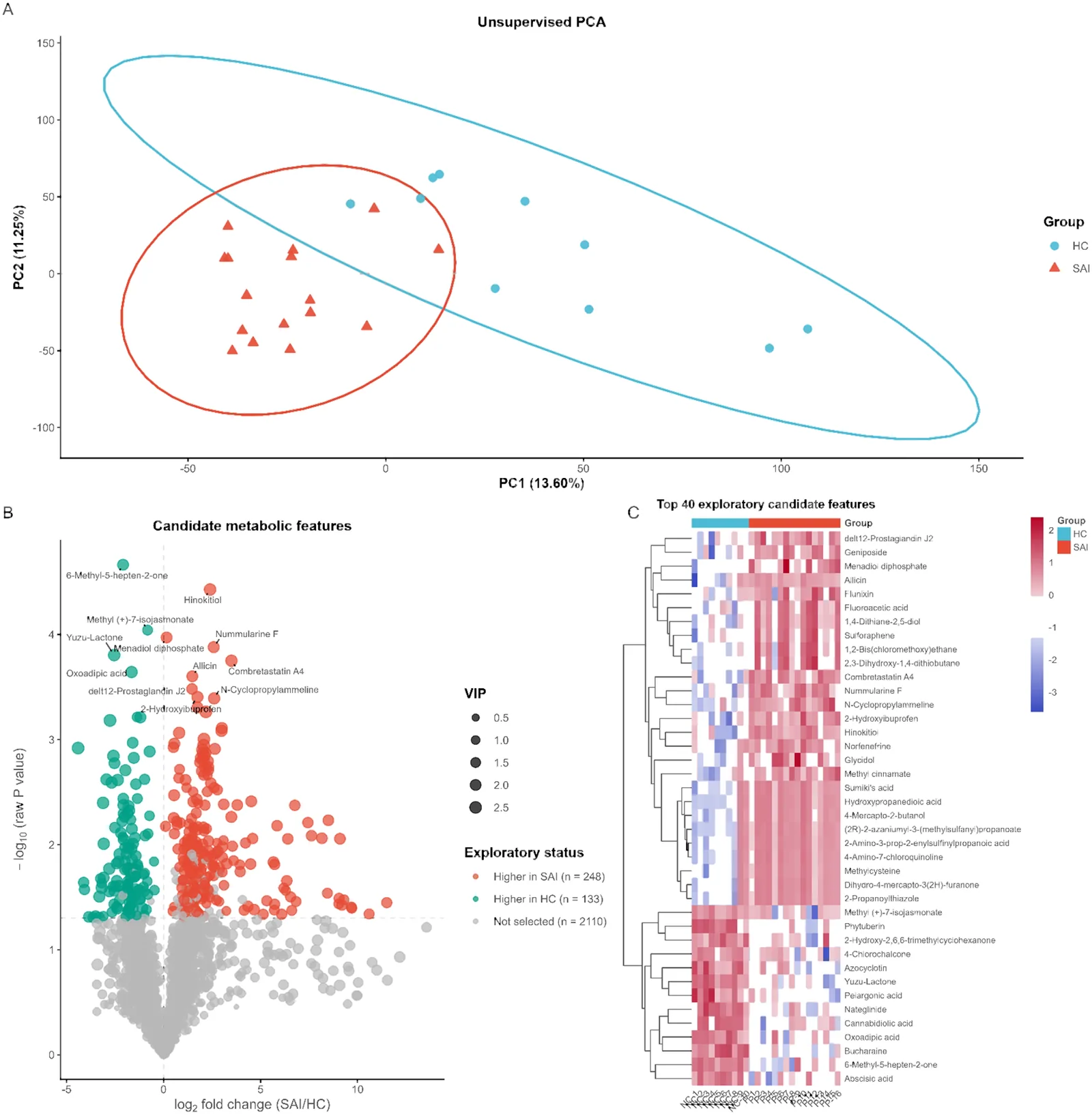
Exploratory fecal metabolomic profiles and candidate metabolic features in the HC and SAI groups. **(A)** Unsupervised principal component analysis of global fecal metabolic profiles. PC1 and PC2 explained 13.60% and 11.25% of the total variation, respectively. Substantial overlap was observed between the HC and SAI samples, together with only a modest trend toward group-associated differentiation. **(B)** Volcano plot of candidate metabolic features screened using VIP > 1 and raw P < 0.05. The x-axis represents log_2_ fold change for the SAI group relative to the HC group, and the y-axis represents −log_10_(raw P value). Point size represents the VIP value. Red points indicate features with relatively higher signals in SAI, green points indicate features with relatively higher signals in HC, and gray points indicate features that did not meet the exploratory screening criteria. **(C)** Clustered heatmap of selected candidate metabolic features. Colors represent row-wise standardized relative signal intensities, with red indicating relatively higher and blue indicating relatively lower signals. The displayed features were selected using exploratory criteria and should not be interpreted as FDR-confirmed metabolic biomarkers. HC, healthy control; SAI, *Staphylococcus aureus* infection; PCA, principal component analysis; VIP, variable importance in projection; FDR, false discovery rate.

Untargeted fecal metabolomics detected 2,956 metabolic features, including 1,520 features in positive-ion mode and 1,436 features in negative-ion mode. A total of 2,491 features received MS/MS-level annotations. Unsupervised PCA showed substantial overlap between the HC and SAI samples, together with only a modest trend toward group-associated differentiation. PC1 and PC2 explained 13.60% and 11.25% of the total variation, respectively (**Figure 4A**). The PCA plot presented in **Figure 4A** was generated in R version 4.6.0 using the processed metabolomic matrix.

An exploratory supervised OPLS-DA model contained one predictive component and one orthogonal component and yielded cumulative R^2^X, cumulative R^2^Y, and cumulative Q^2^ values of 0.185, 0.961, and 0.730, respectively. In the 200-permutation validation, the observed Q^2^ exceeded all permuted Q^2^ values (permutation P < 0.005), whereas the R^2^Y permutation result did not reach statistical significance (P = 0.11). Because OPLS-DA is a supervised method, the sample size was limited, and the permutation metrics did not uniformly support the model, the OPLS-DA result was treated as exploratory and was not regarded as independent confirmatory evidence of group discrimination. The OPLS-DA score plot and permutation-validation plot are presented in **Supplementary Figure 1**. The score plot and permutation-validation panel presented in **Supplementary Figure 1** were generated or assembled in R from the archived OPLS-DA model outputs.

Using the predefined exploratory criteria of VIP > 1 and raw P < 0.05, 381 candidate metabolic features were identified, including 248 features with relatively higher signals and 133 features with relatively lower signals in the SAI group. Because the screening incorporated an unadjusted P-value threshold and several representative features did not remain significant after FDR correction, these findings were interpreted as exploratory candidate metabolic features rather than validated differential metabolites or biomarkers. The volcano plot and clustered heatmap provide descriptive visualizations of these candidate features (**Figures 4B, C**).

Among the representative candidate features, indole-3-lactic acid, indole, serotonin, melatonin, sulfate, pyroglutamic acid, glycine, diethanolamine, and acetylcholine showed relatively higher signals in the SAI group. In contrast, N-methyltryptamine, 6-hydroxymelatonin, acetyl-N-formyl-5-methoxykynurenamine, adenine, adenosine, and glycyl-L-leucine showed relatively higher signals in the HC group (**Table 3**). These observations were interpreted as prioritized exploratory signals requiring independent validation.

**Table 3:** Representative candidate metabolic features identified in the fecal metabolomic analysis.

Candidate metabolic feature	Relative direction	Fold change (SAI/HC)	log2 fold change	VIP	Raw P value	BH-FDR q value
Indole-3-lactic acid	Higher in SAI	3.365	1.751	1.090	0.0108	0.1894
Indole	Higher in SAI	3.385	1.759	1.216	0.0066	0.1760
Serotonin	Higher in SAI	3.187	1.672	1.089	0.0117	0.1911
Melatonin	Higher in SAI	2.719	1.443	1.639	0.0056	0.1691
Sulfate	Higher in SAI	4.995	2.320	1.623	0.0020	0.1100
Pyroglutamic acid	Higher in SAI	2.915	1.544	1.349	0.0188	0.2057
Glycine	Higher in SAI	2.392	1.259	1.367	0.0165	0.2018
Diethanolamine	Higher in SAI	4.209	2.074	1.870	0.0123	0.1913
Acetylcholine	Higher in SAI	4.076	2.027	1.195	0.0267	0.2321
N-Methyltryptamine	Higher in HC	0.250	-2.000	1.977	0.0144	0.1977
6-Hydroxymelatonin	Higher in HC	0.308	-1.699	1.730	0.0058	0.1708
Acetyl-N-formyl-5-methoxykynurenamine	Higher in HC	0.187	-2.419	2.010	0.0151	0.2010
Adenine	Higher in HC	0.207	-2.271	2.249	0.0123	0.1913
Adenosine	Higher in HC	0.247	-2.015	2.039	0.0054	0.1673
Glycyl-L-leucine	Higher in HC	0.228	-2.136	2.003	0.0060	0.1715

Candidate metabolic features shown here are the representative compounds discussed in Section 3.4 and were prioritized using VIP > 1 and raw P < 0.05. Fold change was calculated as SAI/HC; values > 1 indicate relatively higher signals in SAI and values < 1 indicate relatively higher signals in HC. Raw P values are unadjusted; q values were calculated using the Benjamini–Hochberg false discovery rate procedure. None of the listed features remained significant at q < 0.05; therefore, these results are exploratory, hypothesis-generating signals and should not be interpreted as FDR-confirmed biomarkers. Compound identities are putatively annotated from the untargeted LC–MS/MS workflow. BH, Benjamini–Hochberg; FDR, false discovery rate; HC, healthy control; SAI, *Staphylococcus aureus* infection; VIP, variable importance in projection.

### 3.5 Candidate metabolic features show exploratory enrichment in amino acid, sulfur, bile acid, and lipid-related pathways

**Figure 5:**
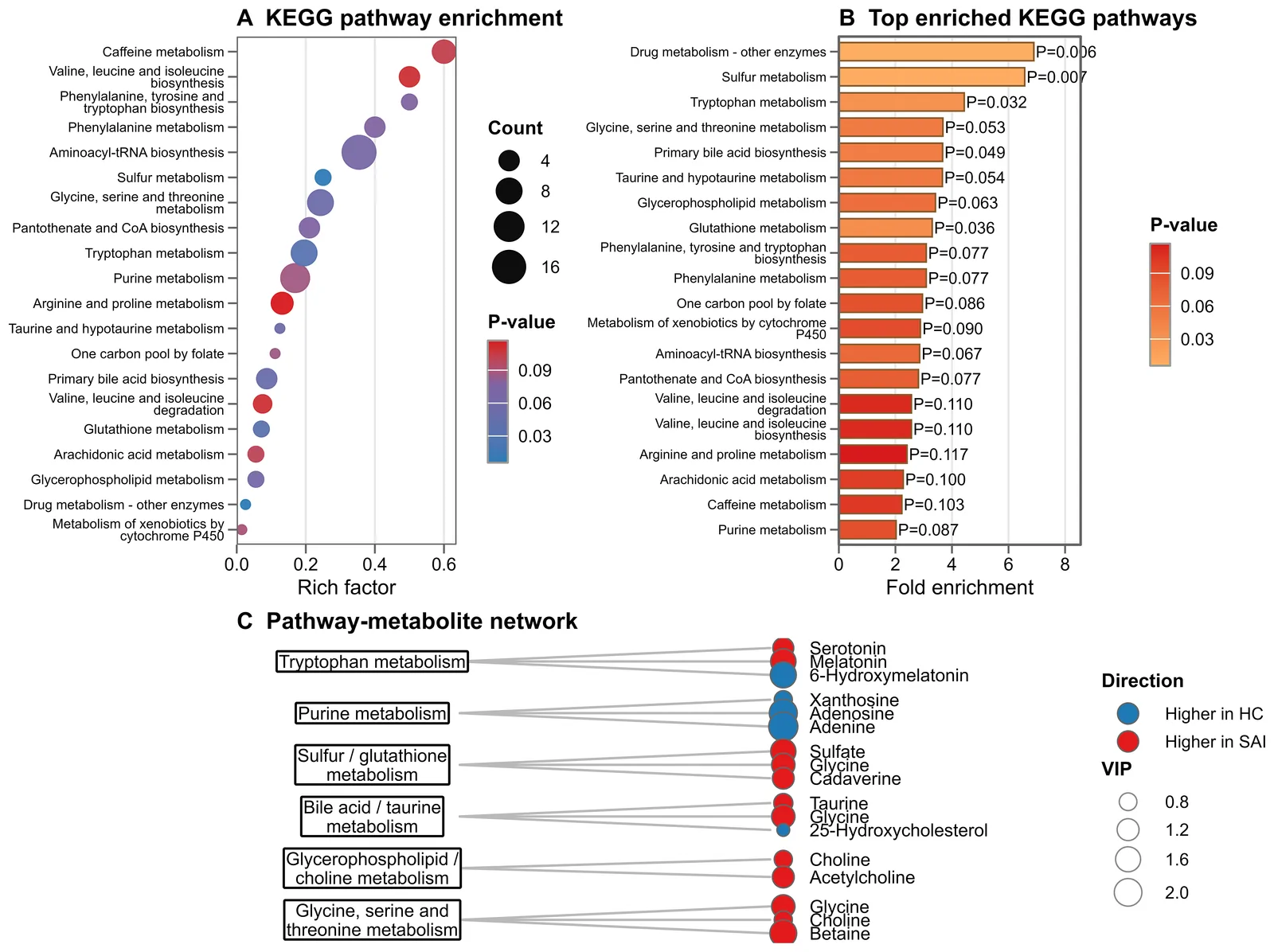
Exploratory metabolomics-based KEGG pathway enrichment and pathway–metabolite network analysis of candidate metabolic features. **(A)** KEGG pathway-enrichment bubble plot based on candidate metabolic features selected using VIP > 1 and raw P < 0.05. The x-axis represents the rich factor, bubble size represents the number of candidate metabolic features assigned to each pathway, and bubble color represents the raw P value. **(B)** Bar plot of selected pathways showing exploratory enrichment. The x-axis represents fold enrichment, and the corresponding raw P values are displayed on the right. **(C)** Pathway–metabolite network linking selected metabolic pathways with representative candidate metabolic features. Red nodes indicate features with relatively higher signals in the SAI group, whereas blue nodes indicate features with relatively higher signals in the HC group. Node size represents the VIP value, and edges indicate annotation of candidate metabolic features to the corresponding pathways. HC, healthy control; SAI, *Staphylococcus aureus* infection; KEGG, Kyoto Encyclopedia of Genes and Genomes; VIP, variable importance in projection; FDR, false discovery rate.

KEGG pathway-enrichment analysis was performed using candidate metabolic features selected using VIP > 1 and raw P < 0.05. Exploratory enrichment signals involved sulfur metabolism, tryptophan metabolism, glycine/serine/threonine metabolism, primary bile acid biosynthesis, taurine and hypotaurine metabolism, glycerophospholipid metabolism, glutathione metabolism, phenylalanine metabolism, and purine metabolism (**Figures 5A, B**).

The pathway–metabolite network linked selected pathways with representative candidate metabolic features, including serotonin, melatonin, 6-hydroxymelatonin, xanthosine, adenosine, adenine, sulfate, glycine, cadaverine, taurine, 25-hydroxycholesterol, choline, acetylcholine, and betaine (**Figure 5C**).

These pathway-enrichment results were derived from candidate metabolic features selected using raw P values and were not interpreted as FDR-confirmed metabolic pathway alterations. The pathways and network were therefore presented only as exploratory, hypothesis-generating patterns for prioritization in future targeted metabolomic and mechanistic studies.

All pathway-enrichment and pathway–metabolite network results were based on metabolic features directly measured using untargeted fecal metabolomics. They did not represent PICRUSt2 output, predicted microbial gene functions, or inferred microbial metabolic capacity.

All pathway-enrichment and pathway–metabolite network analyses were based exclusively on metabolic features directly measured using LC–MS/MS-based untargeted fecal metabolomics. No PICRUSt2-derived output was included in **Figure 5** or used to infer microbial gene functions or microbial metabolic capacity. The displayed candidate features and pathway-enrichment signals were selected using unadjusted P values and were not regarded as FDR-confirmed findings. These results should therefore be interpreted as exploratory and hypothesis-generating rather than as definitive metabolic pathway alterations or predicted microbial functions.

### 3.6 Robust genus-level taxa show exploratory associations with candidate metabolic features

Spearman correlation analysis initially identified 282 genus–metabolite associations meeting the exploratory threshold of |rho| > 0.5 and raw P < 0.05. Pair-specific leave-one-out sensitivity analysis showed that 219 of these associations (77.7%) retained a consistent correlation direction across all valid iterations and continued to meet the predefined threshold in at least 80% of the iterations. The remaining 63 associations did not meet the stability criterion and were excluded from the revised network.

**Figure 6:**
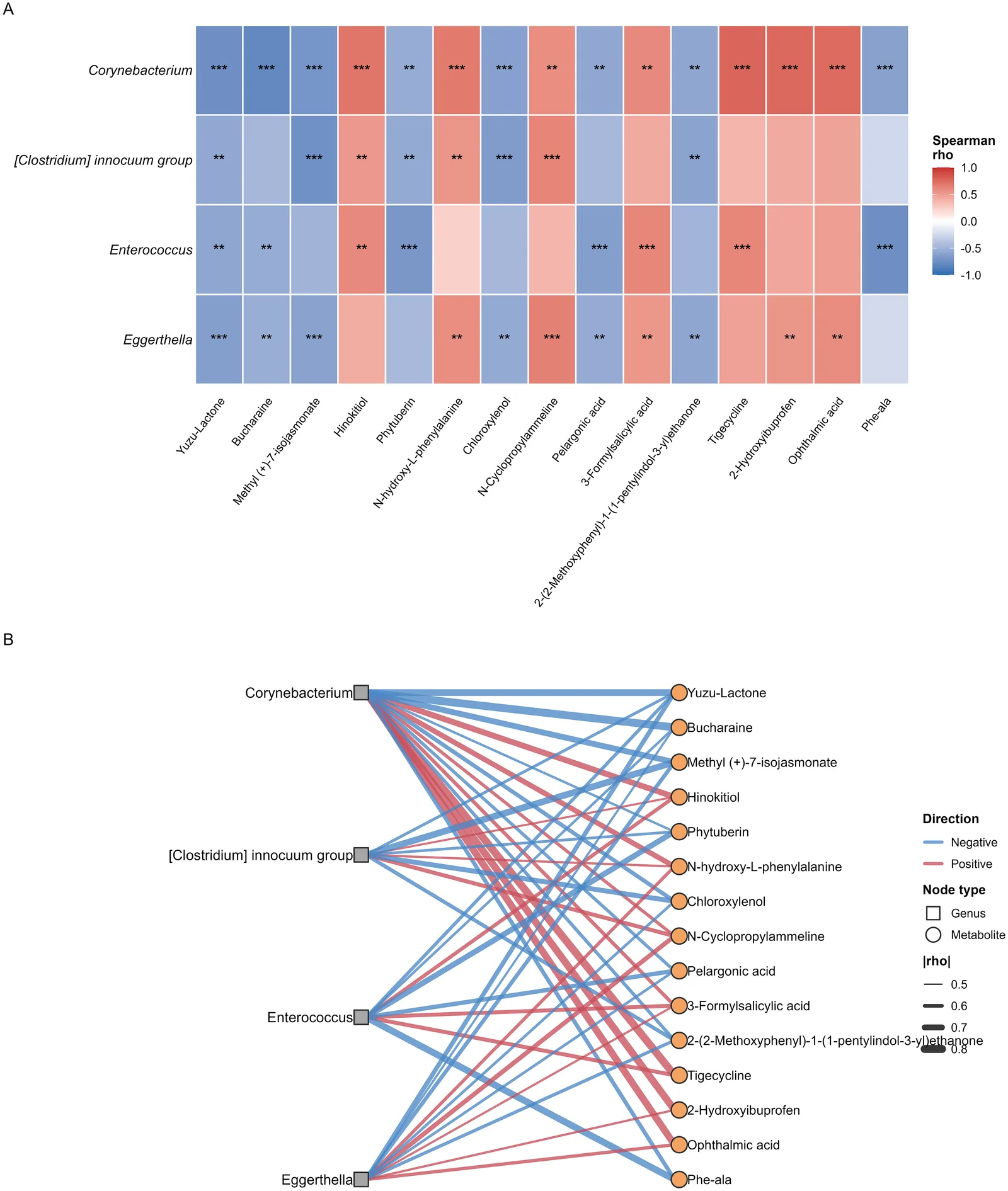
Leave-one-out sensitivity-supported associations between robust genus-level taxa and candidate metabolic features. **(A)** Spearman correlation heatmap between the four robust genus-level taxa identified using ANCOM-BC2 and selected candidate metabolic features. Colors indicate Spearman rho values. Asterisks indicate associations that met the full-data exploratory threshold of |rho| > 0.5 and raw P < 0.05 and passed the predefined leave-one-out sensitivity criterion; the number of asterisks reflects the raw P value (*P < 0.05, **P < 0.01, and ***P < 0.001). **(B)** Exploratory genus–metabolite network displaying selected sensitivity-supported associations involving the metabolic features shown in panel **(A)** Square nodes represent genus-level taxa, circular nodes represent candidate metabolic features, red edges indicate positive correlations, blue edges indicate negative correlations, and edge width represents the absolute Spearman correlation coefficient. The displayed network represents a selected subset of sensitivity-supported associations; complete full-data and leave-one-out results are provided in **Supplementary Table 1**. The associations describe covariance and should not be interpreted as evidence that specific microbial taxa directly produce or regulate the corresponding metabolic features. HC, healthy control; SAI, *Staphylococcus aureus* infection; LOO, leave-one-out.

The revised heatmap and network display selected sensitivity-supported associations involving the four robust genus-level taxa identified using ANCOM-BC2 (**Figures 6A, B**). Complete full-data and leave-one-out results are provided in **Supplementary Table 1**. Although the retained associations were not readily attributable to a single observation, they represent exploratory covariance patterns and do not demonstrate that specific microbial taxa directly produced or regulated the corresponding metabolic features.

Because the genus–metabolite correlations were calculated across the combined HC and SAI sample set, some apparently stable associations may reflect parallel between-group differences rather than within-group biological coupling. In addition, several annotated metabolic features appeared to be medication-related or xenobiotic signals and may reflect treatment exposure rather than endogenous metabolic remodeling. These features were therefore not assigned direct mechanistic interpretations.

### 3.7 Clinical indicators show exploratory covariance with selected omics features

**Figure 7:**
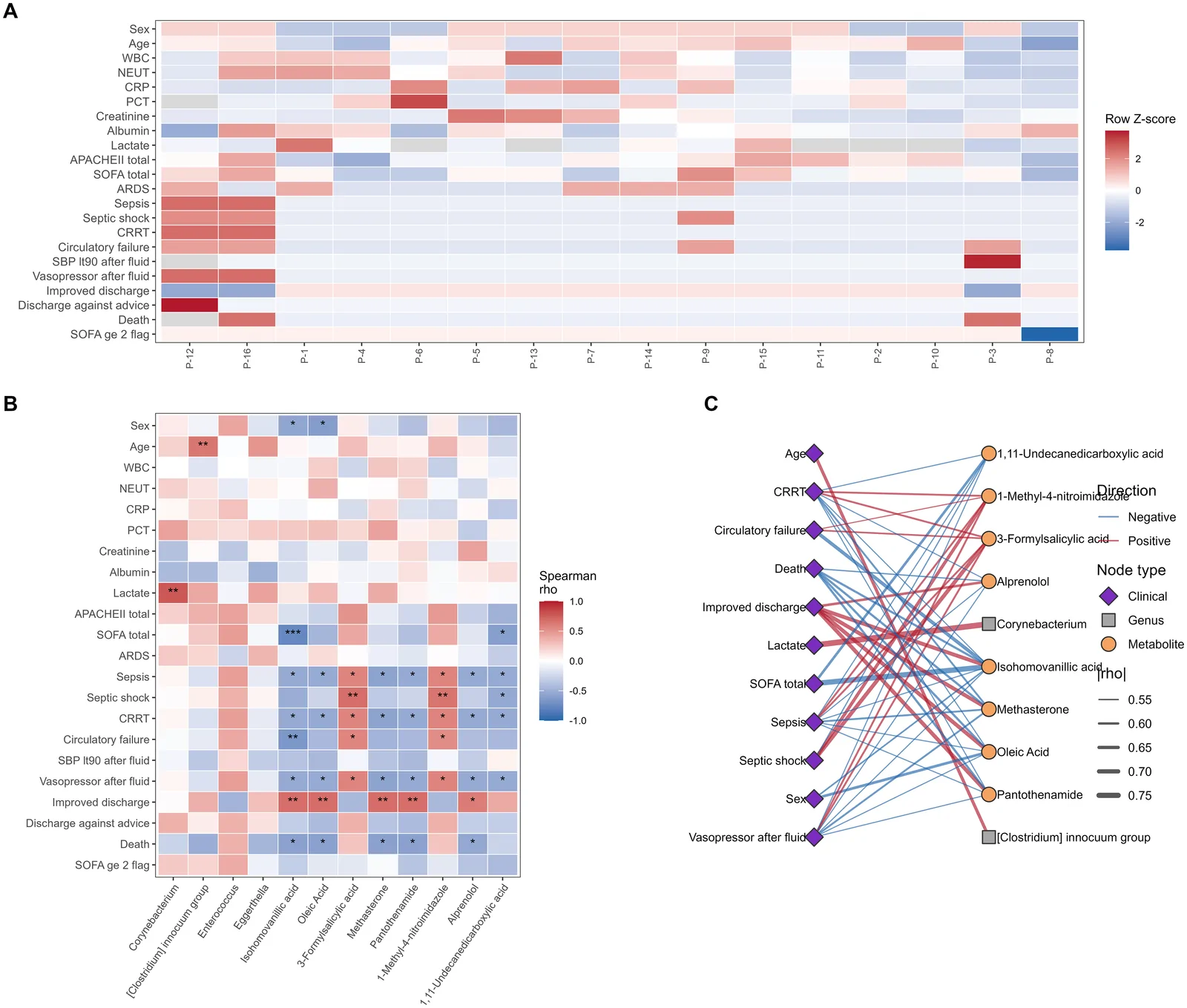
Clinical characteristics and leave-one-out sensitivity-supported clinical–omics associations in patients with SAI. **(A)** Heatmap of clinical indicators in the 16 patients with SAI. Colors represent row-wise standardized values, and gray cells indicate missing observations. **(B)** Spearman correlation heatmap between clinical indicators and selected robust genus-level taxa or candidate metabolic features. Asterisks indicate associations that met the full-data exploratory threshold of |rho| > 0.5 and raw P < 0.05 and passed the predefined leave-one-out sensitivity criterion; the number of asterisks reflects the raw P value (*P < 0.05, **P < 0.01, and ***P < 0.001). **(C)** Exploratory clinical–omics network displaying selected sensitivity-supported associations. Diamond nodes represent clinical indicators, square nodes represent robust genus-level taxa, and circular nodes represent candidate metabolic features. Red edges indicate positive correlations, blue edges indicate negative correlations, and edge width represents the absolute Spearman correlation coefficient. Binary and categorical clinical variables were not subjected to IQR-based outlier screening. None of the clinical–omics associations remained statistically significant after FDR correction. The displayed network represents a selected subset of the sensitivity-supported associations and should be interpreted only as a hypothesis-generating visualization rather than evidence of independent, predictive, mechanistic, or causal relationships. SAI, *Staphylococcus aureus* infection; LOO, leave-one-out; FDR, false discovery rate.

Clinical indicators from the 16 patients with SAI were visualized using a row-standardized heatmap, which demonstrated substantial interindividual heterogeneity in inflammatory markers, organ-function indicators, disease-severity measures, organ-support requirements, and clinical outcomes (**Figure 7A**).

All clinical variables were verified against the source records before the final clinical–omics analysis, and all correlations were recalculated using the corrected dataset.

In the clinical–omics analysis, 481 associations initially met the exploratory threshold of |rho| > 0.5 and raw P < 0.05. Of these, 301 associations (62.6%) met the predefined leave-one-out stability criterion, whereas 180 associations failed the criterion and were excluded from the revised visualization.

The revised clinical–omics heatmap and network display selected sensitivity-supported associations involving clinical indicators, robust genus-level taxa, and candidate metabolic features (**Figures 7B, C**). None of the clinical–omics associations remained statistically significant after Benjamini–Hochberg FDR correction, with the minimum adjusted q value being 0.0609. Complete full-data and leave-one-out results are provided in **Supplementary Table 1**.

Given the limited sample size, sparse binary clinical outcomes, and large number of pairwise comparisons, these associations were interpreted only as hypothesis-generating covariance patterns. They should not be regarded as independent clinical relationships, predictive biomarkers, or evidence of biological causality.

## 4 Discussion

This exploratory study integrated 16S rRNA sequencing, untargeted fecal metabolomics, and clinical phenotype data to characterize gut microbial ecology and fecal metabolic profiles in hospitalized patients with *Staphylococcus aureus* infection. Several principal findings emerged. First, microbial richness and diversity were lower in the SAI group than in the HC group, whereas the overall difference in microbial community composition was statistically significant but modest in magnitude. Second, compositionally aware differential-abundance analysis using ANCOM-BC2 identified four genera with robustly higher bias-corrected abundance in the SAI group: *Corynebacterium*, the *[Clostridium] innocuum* group, *Enterococcus*, and *Eggerthella*. No genus with lower bias-corrected abundance in the SAI group met the predefined robust-significance criterion. Third, 381 exploratory candidate metabolic features were identified using VIP > 1 and raw P < 0.05, with pathway-enrichment signals involving amino acid, sulfur, bile acid, redox-related, and lipid-related pathways. These metabolic findings were not regarded as FDR-confirmed alterations. Fourth, selected microbial, metabolic, and clinical features showed exploratory covariance patterns, although none of the clinical–omics associations remained significant after FDR correction. The directions of the four primary genus-level effect estimates remained positive in analyses restricted to culture-confirmed cases and respiratory tract infections, although not all taxa retained pseudo-count-robust significance in the smaller restricted datasets. Collectively, the findings indicate group-associated differences in gut microbial ecology and the fecal metabolic milieu but do not establish an SAI-specific or causal microbiota–metabolite–host pathway.

Pre-sampling antimicrobial exposure represents the most important potential confounder in interpreting the present findings. Fifteen of the 16 patients with SAI had received antimicrobial treatment before fecal sample collection, whereas one patient had not received antimicrobial treatment before sampling. None of the healthy controls had received antibiotics before fecal collection. Although the temporal ordering of antimicrobial administration and fecal sampling was confirmed, the exact treatment-to-sampling interval, treatment duration, dosage, and cumulative pre-sampling exposure could not be consistently quantified.

Because almost all patients with SAI were sampled after antimicrobial exposure, whereas all healthy controls were antimicrobial-unexposed before sampling, the observed microbial and metabolic differences cannot be separated from the potential effects of antimicrobial treatment. Hospitalization, disease severity, altered nutritional intake, glucocorticoid exposure, organ-support measures, underlying diseases, and other clinical interventions may also have contributed to the observed patterns. The observed reduction in microbial diversity and the higher bias-corrected abundance of Enterococcus and the other ANCOM-BC2-supported genera should therefore be interpreted as an intestinal ecological phenotype observed in hospitalized patients with SAI rather than as a pathogen-specific effect caused by *S. aureus*. Secondary exploratory decreases in selected commensal taxa were not FDR-confirmed and were not used for primary inference. This cautious interpretation is consistent with previous evidence showing that intestinal dysbiosis during severe infection may reflect the combined effects of antimicrobial treatment, systemic inflammation, hospitalization, nutritional alteration, organ dysfunction, and critical illness rather than the effect of a single pathogen (Sun et al., 2023; Piccioni et al., 2024; Tao et al., 2024). In the specific context of *S. aureus*, current evidence linking infection with intestinal microbial alterations remains limited, and recent literature has emphasized the importance of distinguishing pathogen-associated effects from host-related and treatment-related influences (Liu et al., 2024).

The infection-confirmation sensitivity analyses provided additional support for the directional stability of the primary genus-level findings. All four bias-corrected effect estimates remained positive after restriction to culture-confirmed cases and after restriction to respiratory tract infections, indicating that the primary directions were not driven solely by the BALF sequencing-confirmed cases or by the two bloodstream-infection cases. Nevertheless, the robustness of individual taxa was not uniform across the restricted datasets. In the culture-confirmed subset, the *Enterococcus* and *Eggerthella* estimates did not pass pseudo-count sensitivity, whereas in the respiratory-infection-only subset, the *Eggerthella* estimate remained sensitive to pseudo-count addition. These findings support directional consistency of the four genus-level signals but also indicate greater statistical uncertainty for some taxa when the analysis was restricted to smaller clinical subsets.

The higher bias-corrected abundance of Enterococcus in the SAI group is biologically plausible in hospitalized and antimicrobial-exposed populations. Antibiotic treatment can reduce colonization resistance and facilitate intestinal expansion of enterococci, particularly in medically complex patients (Ubeda et al., 2010; Arias and Murray, 2012). However, the present data cannot determine whether the observed Enterococcus pattern preceded infection, resulted from antimicrobial exposure, reflected hospitalization-related ecological disruption, or represented a combination of these factors. Similarly, the higher abundance of *Corynebacterium*, *[Clostridium] innocuum* group, and *Eggerthella* should not be interpreted as direct evidence that these taxa contribute to *S. aureus* pathogenesis. Their identification should instead be regarded as a reproducible statistical signal within this small cohort that warrants evaluation in larger, better-controlled studies.

The untargeted metabolomic analysis identified candidate features involving tryptophan-associated, redox-related, bile-acid-related, sulfur-related, and lipid-related pathways. Nevertheless, these candidate features were screened using VIP > 1 and raw P < 0.05, and the highlighted pathway-enrichment findings were not regarded as FDR-confirmed alterations. Therefore, these metabolic results should be interpreted as prioritized exploratory signals rather than validated biomarkers or definitive pathway abnormalities.

Several representative candidate features, including serotonin, melatonin, indole-related compounds, glycine, sulfate, choline-related compounds, adenine, and adenosine, may originate from or be influenced by multiple sources. These sources include host metabolism, microbial metabolism, diet, hepatic and renal function, intestinal transit, medication exposure, and other environmental factors. Detailed dietary intake and non-antimicrobial concomitant medication exposure were not systematically recorded in this cohort. In particular, serotonin- and melatonin-related signals cannot be attributed specifically to *S. aureus* infection or to a defined microbial pathway. Similarly, several annotated metabolic features appeared to be medication-related or xenobiotic signals and may reflect treatment exposure rather than endogenous metabolic remodeling.

The exploratory enrichment of tryptophan-related pathways is of potential interest because intestinal microorganisms and host tissues both contribute to tryptophan metabolism. Microbial indole derivatives can influence epithelial-barrier function and mucosal immune responses, whereas host kynurenine and serotonin pathways are associated with inflammation, neurological signaling, and systemic metabolism (Agus et al., 2018; Roager and Licht, 2018; Zelante et al., 2013). However, the present cross-sectional data cannot determine the biological source of individual tryptophan-related features or whether the observed differences were driven by infection, medications, diet, altered intestinal transit, or other clinical factors.

Exploratory signals involving glutathione and sulfur metabolism may be related to changes in redox balance, whereas bile-acid-related and glycerophospholipid-related features may reflect altered intestinal, hepatic, microbial, or inflammatory metabolism. Bile acids participate in lipid absorption and also regulate immune and metabolic processes through receptor-mediated pathways, including farnesoid X receptor and Takeda G protein-coupled receptor 5 signaling (Ridlon et al., 2006; Wahlström et al., 2016). Glycerophospholipid- and choline-related features may reflect membrane turnover, inflammatory lipid signaling, dietary exposure, or medication-related effects. These interpretations remain speculative because the pathway analyses were based on candidate features selected using unadjusted P values and were not supported by targeted metabolomic confirmation.

A key feature of this study is the integration of paired microbial-composition and fecal-metabolomic data with clinical phenotypes. Pair-specific leave-one-out analyses showed that 219 of 282 initially selected genus–metabolite associations and 301 of 481 initially selected clinical–omics associations met the predefined stability criterion. However, leave-one-out stability does not establish statistical independence, external reproducibility, biological mechanism, or causality. None of the clinical–omics associations remained statistically significant after FDR correction, with the minimum adjusted q value being 0.0609. In addition, the genus–metabolite correlations were calculated across the combined HC and SAI sample set, meaning that some apparently stable associations may reflect parallel between-group differences rather than within-group biological coupling.

The retained correlations should therefore be interpreted only as exploratory covariance patterns. They do not demonstrate that specific microbial taxa produced, consumed, or regulated individual metabolic features, nor do they show that the corresponding features directly influenced inflammatory or organ-function indicators. These covariance patterns provide hypotheses regarding possible relationships among intestinal microbial ecology, fecal metabolic features, and clinical phenotypes. However, the cross-sectional design and extensive treatment-related confounding do not allow determination of whether the observed intestinal changes contributed to the host response or were consequences of infection, hospitalization, treatment exposure, or other clinical factors.

The present study has several strengths. The use of paired fecal samples for 16S rRNA sequencing and untargeted metabolomics improved correspondence between the microbial and metabolic datasets. The primary genus-level analysis was revised using ANCOM-BC2 with FDR correction and pseudo-count sensitivity analysis, reducing reliance on unadjusted relative-abundance comparisons. PERMDISP was performed alongside PERMANOVA to assess whether the beta-diversity result could be explained by unequal group dispersion. The supervised OPLS-DA analysis was evaluated using permutation testing and was retained only as a supplementary exploratory visualization. Pair-specific leave-one-out analyses were also used to assess whether selected correlation signals were disproportionately influenced by individual observations. Together, these analyses improved the transparency and robustness of the exploratory statistical framework.

Several limitations should be acknowledged. First, this was a single-center cross-sectional study with a small sample size, which limited statistical power, increased susceptibility to interindividual variability, and prevented robust multivariable adjustment. Age and sex were available for both groups, and no statistically significant between-group differences were detected. However, the limited sample size reduced the ability to exclude clinically meaningful demographic imbalance. Detailed information regarding body mass index, chronic comorbidities, dietary intake, and non-antimicrobial concomitant medications was not systematically available for the healthy controls. Therefore, residual confounding by these and other unmeasured baseline characteristics could not be fully evaluated or adjusted for. The importance of accounting for these variables is supported by evidence that host demographic, lifestyle, physiological, and clinical characteristics can substantially confound human gut microbiota studies (Vujkovic-Cvijin et al., 2020).

Second, pre-sampling antimicrobial exposure differed substantially between groups. Fifteen of the 16 patients with SAI had received antimicrobial treatment before fecal collection, whereas none of the healthy controls had received antibiotics before sampling. Excluding the only antimicrobial-unexposed patient in a limited sensitivity analysis did not resolve this confounding because all 15 remaining patients with SAI were antimicrobial-exposed. Although the sequence of antimicrobial administration and fecal sampling was confirmed, the exact treatment-to-sampling interval, treatment duration, dosage, and cumulative pre-sampling exposure could not be consistently quantified. Consequently, the observed microbiome and metabolomic patterns cannot be separated from the potential effects of antimicrobial treatment.

Third, fecal samples from patients with SAI were collected within 7 days after microbiological confirmation rather than on an identical prespecified day. Fecal-sampling timestamps were recorded and were used to define the 24-h clinical-data window. However, because sampling depended on spontaneous bowel movements, the exact elapsed interval between microbiological confirmation and fecal collection was not available as a sufficiently complete and standardized patient-level variable for quantitative analysis. Temporal variation in fecal collection may therefore have contributed to microbial and metabolic heterogeneity.

Fourth, the original genus-level relative-abundance analysis was exploratory. A compositionally aware method with FDR correction and pseudo-count sensitivity analysis was therefore adopted as the primary revised analysis. Taxa identified only through raw P values in the secondary STAMP analysis should not be interpreted as validated differential taxa. Although the directions of the four primary genus-level estimates remained consistent in both infection-related sensitivity analyses, not all taxa retained pseudo-count-robust significance in the smaller restricted datasets. In addition, PERMDISP was statistically significant in the culture-confirmed subset, indicating that its significant PERMANOVA result may have been influenced partly by unequal multivariate dispersion. The culture-confirmed beta-diversity result should therefore be interpreted cautiously.

Fifth, the candidate metabolic features and pathway-enrichment signals were selected using VIP > 1 and raw P < 0.05. The highlighted metabolic features and pathways were not regarded as FDR-confirmed findings. Dietary intake, non-antimicrobial medications, and potential exogenous exposures were incompletely documented and may have influenced several annotated features, particularly those related to serotonin, melatonin, bile acids, xenobiotics, and lipid metabolism. Targeted quantitative validation was not performed.

Sixth, the PCoA and metabolomic PCA plots showed substantial overlap between the HC and SAI samples. Study group explained approximately 10.7% of the Bray–Curtis microbial community variation. Although PERMDISP did not identify a statistically significant difference in dispersion, the modest PERMANOVA effect size limits the strength of group-discrimination claims. The OPLS-DA model was evaluated using 200 random-label permutations; however, because OPLS-DA is supervised, the sample size was limited, and the R^2^Y permutation result did not reach statistical significance, the model remained susceptible to overfitting and was retained only as a supplementary exploratory analysis.

Seventh, the sequencing provider generated PICRUSt2-based inferred functional profiles as part of its standard deliverables, but these outputs were not included in the analytical framework, figures, tables, statistical analyses, or biological interpretation of the present study. All pathway-enrichment findings reported in the manuscript were derived exclusively from metabolic features directly measured using untargeted fecal metabolomics. They should not be interpreted as predicted microbial gene functions or inferred microbial metabolic capacity.

Eighth, 16S rRNA sequencing does not provide strain-level resolution or direct functional-gene information and cannot determine the biological source of individual metabolic features. Pair-specific leave-one-out analyses improved assessment of sensitivity to individual observations, but 63 of 282 initially selected genus–metabolite associations and 180 of 481 initially selected clinical–omics associations failed the predefined stability criterion. None of the clinical–omics associations remained significant after FDR correction. Correlation and network analyses therefore describe exploratory covariance patterns and cannot demonstrate that particular microbial taxa produce or regulate specific metabolic features or directly influence clinical outcomes.

Future studies should include larger multicenter cohorts, well-characterized and appropriately comparable control groups, longitudinal sampling at standardized time points, detailed documentation of antimicrobial and non-antimicrobial treatment exposure, dietary assessment, targeted quantitative metabolomics, shotgun metagenomic sequencing, microbial-culture experiments, and functional validation. Such studies will be required to distinguish infection-associated signals from hospitalization-related and treatment-related effects and to determine whether any microbial or metabolic feature has independent clinical or mechanistic relevance.

## 5 Conclusions

Based on paired 16S rRNA sequencing and untargeted fecal metabolomics, this exploratory study identified group-associated differences in gut microbial composition and fecal metabolic profiles between hospitalized patients with SAI and healthy controls. Four genera showed robustly higher bias-corrected abundance in the SAI group in the primary ANCOM-BC2 analysis. The directions of these genus-level effect estimates remained positive in analyses restricted to culture-confirmed cases and respiratory tract infections, although some taxa did not retain pseudo-count-robust significance in the smaller restricted datasets.

Candidate metabolic features involved tryptophan-related, glutathione-related, sulfur-related, bile-acid-related, amino-acid-related, and lipid-related pathways. However, these candidate features and pathway-enrichment signals were identified using VIP > 1 and raw P < 0.05 and were not regarded as FDR-confirmed findings. Integrated microbiome–metabolome and clinical–omics associations were also exploratory, and none of the clinical–omics associations remained significant after FDR correction.

Fifteen of the 16 patients with SAI had received antimicrobial treatment before fecal sampling, whereas none of the healthy controls had received antibiotics before sampling. The observed microbial and metabolic patterns therefore cannot be separated from the effects of antimicrobial treatment, hospitalization, disease severity, and other clinical interventions. The findings should be interpreted as preliminary, treatment-context, group-associated observations rather than as alterations caused specifically by *S. aureus*. Larger longitudinal studies incorporating standardized pretreatment and post-treatment sampling, detailed treatment-exposure records, appropriately comparable hospitalized controls, targeted metabolomic validation, metagenomic sequencing, and functional experiments are required.

## Data Availability

The datasets presented in this study are available in online repositories and in the article/Supplementary Material. The 16S rRNA gene amplicon sequencing data have been deposited in the NCBI Sequence Read Archive under BioProject accession PRJNA1507566 (https://www.ncbi.nlm.nih.gov/sra/PRJNA1507566). Processed metabolomic data, de-identified participant metadata, and the other data supporting the findings of this study are included in Supplementary Table 1. The raw untargeted metabolomics data supporting the conclusions of this article will be made available by the corresponding authors upon reasonable request, without undue reservation. Further inquiries can be directed to the corresponding authors.

## Supplementary material

Supplementary Table 1 Multi-sheet Excel workbook containing de-identified participant metadata, healthy-control demographic information, patient-level clinical and infection characteristics, pre-sampling antimicrobial exposure, 16S rRNA sequencing quality-control metrics, sample-level alpha-diversity indices, primary ANCOM-BC2 results, secondary exploratory relative-abundance results, candidate metabolic-feature data, pathway-enrichment results, full correlation and leave-one-out sensitivity results, infection-related sensitivity analyses, and software-version information.

Supplementary Figure 1 Exploratory OPLS-DA score plot and corresponding 200-permutation validation.

Supplementary Figure 2 Sensitivity analyses of the four primary ANCOM-BC2 genus-level effect estimates after restriction to culture-confirmed cases and respiratory tract infections.
