# Supplementary material for: Gut microbiota and fecal metabolomic alterations in hospitalized patients with Staphylococcus aureus infection: an exploratory multi-omics study
Source: Front Cell Infect Microbiol. 2026 Sep 3;16:1919715. doi: 10.3389/fcimb.2026.1919715 (PMC13581951; doi:10.3389/fcimb.2026.1919715)
Supplement: Supplementary file 1 [file Table2.docx]

**Supplementary Figure S1. Exploratory OPLS-DA model and permutation validation of fecal metabolic profiles.**

(A) Exploratory OPLS-DA score plot of fecal metabolic profiles in the HC and SAI groups. The model contained one predictive component and one orthogonal component.
(B) Validation of the OPLS-DA model using 200 random-label permutations. The original model had cumulative R²X = 0.185, cumulative R²Y = 0.961, and cumulative Q² = 0.730. The observed Q² exceeded all permuted Q² values, corresponding to a permutation P value < 0.005, whereas the R²Y permutation P value was 0.11. Because OPLS-DA is supervised, the sample size was limited, and the permutation metrics did not uniformly support the model, the analysis was treated as exploratory and was not used as independent confirmatory evidence of group discrimination. HC, healthy control; SAI, *Staphylococcus aureus* infection; OPLS-DA, orthogonal partial least squares discriminant analysis.

Supplementary Figure S2. Infection-confirmation and infection-site sensitivity analyses of the four primary genera.

Bias-corrected ANCOM-BC2 log fold-change estimates and 95% confidence intervals are shown for the primary analysis including all 16 patients with SAI, the analysis restricted to 12 culture-confirmed cases, and the analysis restricted to 14 patients with respiratory tract infection. Positive values indicate higher estimated abundance in the SAI group relative to the HC group. All four effect estimates retained the same direction across the three analyses. In the culture-confirmed subset, *Enterococcus* and *Eggerthella* did not pass pseudo-count sensitivity. In the respiratory-infection-only subset, *Eggerthella* did not pass pseudo-count sensitivity. HC, healthy control; SAI, Staphylococcus aureus infection; CI, confidence interval.
